# *Acinetobacter* type VI secretion system comprises a non-canonical membrane complex

**DOI:** 10.1371/journal.ppat.1011687

**Published:** 2023-09-28

**Authors:** Ona Kandolo, Yassine Cherrak, Isaac Filella-Merce, Hugo Le Guenno, Artemis Kosta, Leon Espinosa, Pierre Santucci, Christophe Verthuy, Régine Lebrun, Michael Nilges, Riccardo Pellarin, Eric Durand

**Affiliations:** 1 Laboratoire d’Ingénierie des Systèmes Macromoléculaires (LISM), Institut de Microbiologie, Bioénergies and Biotechnologie (IM2B), Aix-Marseille Université, Centre National de la Recherche Scientifique (CNRS)-UMR 7255, Marseille, France; 2 Institut Pasteur, Structural Bioinformatics Unit, Department of Structural Biology and Chemistry, Paris, France; 3 Sorbonne Université, Collège doctoral, Paris, France; 4 Microscopy Core Facility, Aix Marseille Univ, CNRS, Institut de Microbiologie de la Méditerranée, Marseille Cedex 20, France; 5 Laboratoire de Chimie Bactérienne (LCB), Institut de Microbiologie, Bioénergies and Biotechnologie (IM2B), Aix-Marseille Université, Centre National de la Recherche Scientifique, Marseille, France; 6 Proteomic Core Facility IMM, Marseille Protéomique (MaP), Aix Marseille Univ, Marseille Cedex 20, France; 7 Laboratoire d’Ingénierie des Systèmes Macromoléculaires (LISM), Institut de Microbiologie, Bioénergies and Biotechnologie (IM2B), Aix-Marseille Université, Institut National de la Santé et de la Recherche Médicale (INSERM), Marseille, France; Gifu University, JAPAN

## Abstract

*A*. *baumannii* can rapidly acquire new resistance mechanisms and persist on abiotic surface, enabling the colonization of asymptomatic human host. In *Acinetobacter* the type VI secretion system (T6SS) is involved in twitching, surface motility and is used for interbacterial competition allowing the bacteria to uptake DNA. *A*. *baumannii* possesses a T6SS that has been well studied for its regulation and specific activity, but little is known concerning its assembly and architecture. The T6SS nanomachine is built from three architectural sub-complexes. Unlike the baseplate (BP) and the tail-tube complex (TTC), which are inherited from bacteriophages, the membrane complex (MC) originates from bacteria. The MC is the most external part of the T6SS and, as such, is subjected to evolution and adaptation. One unanswered question on the MC is how such a gigantesque molecular edifice is inserted and crosses the bacterial cell envelope. The *A*. *baumannii* MC lacks an essential component, the TssJ lipoprotein, which anchors the MC to the outer membrane. In this work, we studied how *A*. *baumannii* compensates the absence of a TssJ. We have characterized for the first time the *A*. *baumannii’*s specific T6SS MC, its unique characteristic, its membrane localization, and assembly dynamics. We also defined its composition, demonstrating that its biogenesis employs three *Acinetobacter*-specific envelope-associated proteins that define an intricate network leading to the assembly of a five-proteins membrane super-complex. Our data suggest that *A*. *baumannii* has divided the function of TssJ by (1) co-opting a new protein TsmK that stabilizes the MC and by (2) evolving a new domain in TssM for homo-oligomerization, a prerequisite to build the T6SS channel. We believe that the atypical species-specific features we report in this study will have profound implication in our understanding of the assembly and evolutionary diversity of different T6SSs, that warrants future investigation.

## Author summary

Assembly of envelope-spanning secretion nanomachines requires the concerted action of architectural proteins whose conservation have helped their identification in bacterial genomes. Specific adaptation occurs to fine tune the nanomachine to specific bacterial environment or biology. The type VI secretion system (T6SS) assembles a trans-envelope channel, the membrane complex (MC) that plays a key role in the transport of the toxin-loaded speargun. Even though the evolutionary pressure has maintained a high degree of conservation of this complex across species, here we demonstrated that *Acinetobacter baumannii* has evolved two new players to compensate the absence of the highly conserved TssJ lipoprotein. Our finding improves our understanding of the key roles of the lipoprotein: MC stability and multimerization. Understanding the molecular evolution of nanomachines offers the possibility to better understand their architecture and functioning, but also for pathogenic bacteria to identify specific drug target to block their virulence.

## Introduction

The type VI secretion system (T6SS) is a machinery commonly used by 25% of Gram-negative bacteria [1] to inject toxic effectors in the cytoplasm of neighbor cells thus facilitating nutrient access in bacterial niches. In addition to its function in bacterial competition and niche colonization, the T6SS has been shown to play a key role in pathogenesis and virulence. In *Acinetobacter* the T6SS is used for interbacterial competition, targeting both monoderm and diderm bacteria [2,3], and allows the bacteria to uptake DNA [4,5]. The T6SS is also involved in *A*. *baumannii* in twitching and surface motility [6]. A regulatory plasmid is present in a high number of clinical strains to switch-off the machinery under certain stress conditions [7,8].

**Fig 1:**
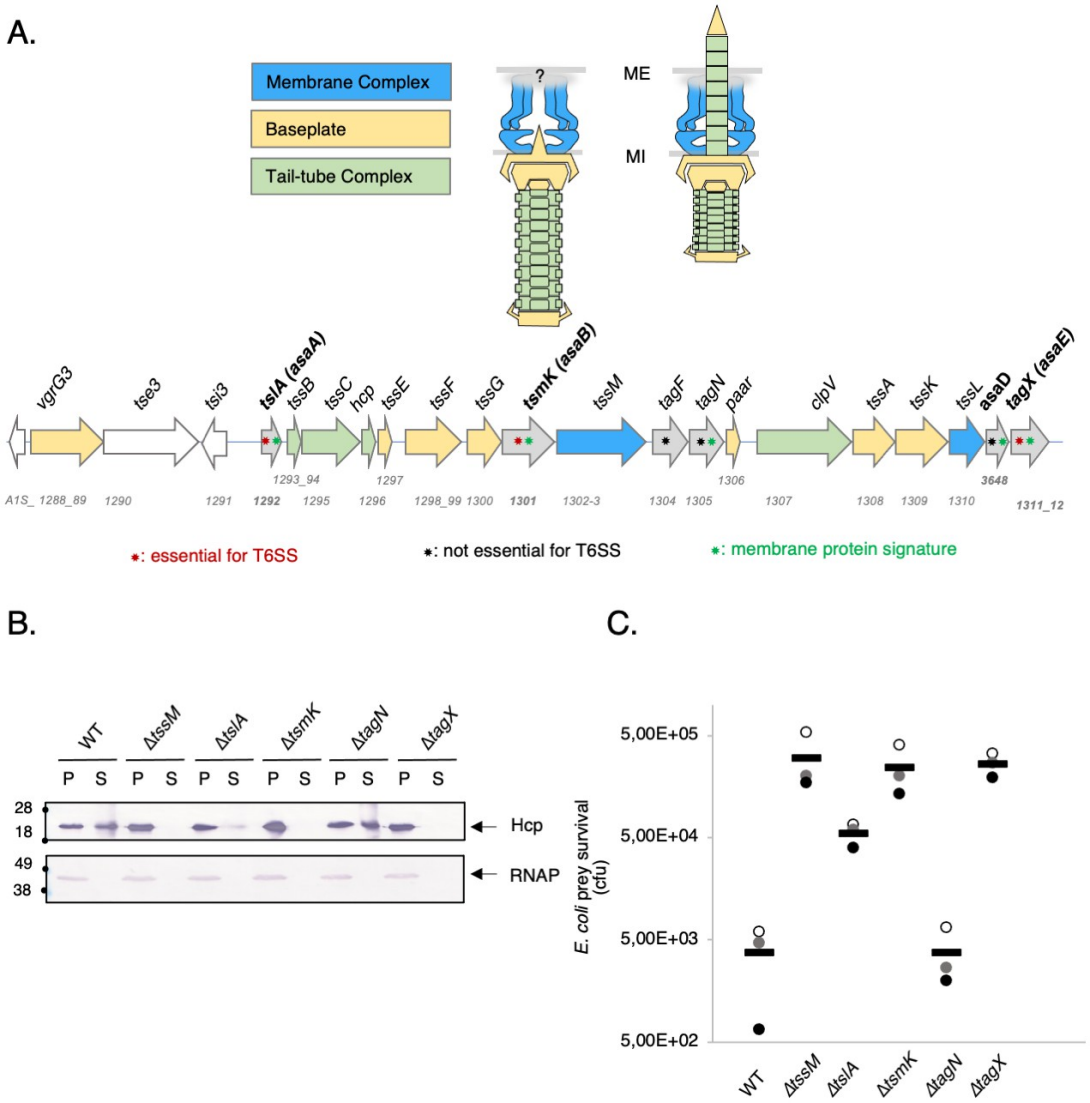
The *A. baumannii* T6SS gene cluster: Systematic review of genes important for the T6SS activity. **(A)** Schematic model of *A*. *baumannii* T6SS nanomachine and representation of the genetic organization of a *vgrG* locus and the T6SS cluster from *A*. *baumannii* 17978. The T6SS gene cluster of *A*. *baumannii* ATCC 17978 covers a 23 kb region that includes 18 putative genes predicted to encode for T6SS components. Genes encoding the Membrane Complex (MC), Tube Complex Tail (TTC), and the Baseplate (BP) in blue, yellow and green respectively. T6SS associated genes are represented in grey. 4 proteins encoded by genes only identified in *A*. *baumannii* T6SS gene clusters (asa, *Acinetobacter* type six secretion system-associated, in bold). According to our results and previous studies, some of those proteins have a membrane protein signature (green stars), are essential for the T6SS functionality (red stars), and others are not essential (black stars). **(B)** Western blot assays probing for Hcp secretion and RNA polymerase (RNAP) in whole-cell pellets (P) and supernatants (S), by *A*. *baumannii* ATCC 17978 wildtype (WT, parental strain) and several mutants. The RNAP was used as lysis control. The supernatants of each strain of *A*. *baumannii* are isolated, concentrated and then analyzed by denatured 12.5%-polyacrylamide gel electrophoresis (PAGE). Immunodetected proteins are indicated on the right. Molecular weight markers (in kDa) are indicated on the left. **(C)** Bacterial competition experiments. Survival of *E*. *coli* rifampicin-resistant after incubation with ATCC 17978 WT and several mutants. The *A*. *baumannii* predator strains indicated are on the x axis and the log-transformed surviving *E*. *coli* CFU count is on the y axis. Bars are mean values and dots are three independent replicates.

The T6SS is a contractile nanomachine, structurally and evolutionarily related to the tail of the T4 bacteriophage [9,10]. This double membrane spanning structure assembles in three major complexes: the Membrane Complex (MC), the Baseplate (BP) and the Tail-Tube Complex (TTC) [11,12]. The T6SS is assembled by 13 to 15 core proteins (*tss*) which are essential for the T6SS functionality. They are organized in single or multiple genetic clusters, which also contain additional genes (*tag*), encoding accessory proteins often with unknown functions [13–15]. Species-specific adaptations have been described for *Vibrio fischeri* (TasL, [16]) or *Pseudomonas aeruginosa* (TagJ, [17,18]) that fine-tune the assembly and/or architecture of the T6SS to specific demand. Among the eighteen genes of the *Acinetobacter* T6SS cluster, fourteen have a homology with T6SS components in other bacteria. Previous studies have demonstrated the existence of specific components and the remarkable absence of the highly conserved component TssJ [14,15]. The TssJ lipoprotein is a key and conserved component of the MC in other bacteria that plays a central role in the nucleation, assembly and outer membrane insertion of the MC [19,20]. In *A*. *baumannii* model strain ATCC 17978 TssL (*A1S_1310* locus) and TssM (*A1S_1302_03* locus) are the well described and canonical components of the T6SS MC [21] (Fig 1A). In addition, four genes (*A1S_1292*, *A1S_1301*, *A1S_3648* and *A1S_1311_12* loci) in the T6SS cluster encode membrane-associated proteins. Since they are unique to *A*. *baumannii*, these proteins have been called **Asa**, (***A****cinetobacter* type **s**ix secretion system-**a**ssociated) [14]. *A1S_1292* encodes a periplasmic protein (AsaA or TslA) capable of interacting with TssM [22] and crucial for T6SS assembly at the cell-cell contact [23]. *A1S_1311_12* encodes a polytopic membrane protein (AsaE or TagX) with a lytic transglycosylase activity that enables the transit of the T6SS trans-envelope complex across the peptidoglycan layer of the T6SS-producing bacterium [7]. Until now, A1S_1301 (AsaB) has never been characterized and no connection has been established between these accessory proteins, neither if they participate to the assembly of the MC.

The stabilization exerted by TssJ on the T6SS MC prompts the question of how MC assembly occurs in T6SSs containing no identified anchoring protein. More broadly, apart for its structural role, little is known about the specific function of the TssJ lipoprotein during T6SS functioning. By studying the unconventional *A*. *baumannii* MC that is devoid of a TssJ homolog we might answer these questions. Studying the *A*. *baumannii* T6SS MC challenges our current view of this central pillar in the T6SS nanomachine and offers a unique glimpse of specific adaptation to particular bacterial lifestyle.

In the present study, we monitored and characterized the composition and assembly of the *A*. *baumannii* T6SS MC. We notably demonstrated the existence of an intricate protein-protein interaction network encompassing 5 membrane or membrane-associated proteins. Our work revealed that the assembly of the *A*. *baumannii*-specific MC is orchestrated by an uncharacterized protein encoded by the gene *asaB* that we renamed TsmK (**T**ype **s**ix secretion **m**embrane complex assembly pseudo-**K**etoacyl synthase). We gathered several lines of evidence on the function of TsmK in MC assembly, particularly its stabilizing effect and its predicted structure. Our study highlights the presence of a Glycine-Serine linker conserved in the C-terminus of *Acinetobacter* TssM and that is crucial to the functioning of the T6SS. Finally, our sequence analysis reveals a conditional dependency on the presence of TsmK, the “GS-linker” of TssM and the absence of TssJ-homologs as a feature specific to *Acinetobacter* T6SS, elements that altogether might compensate the absence of a TssJ homolog.

## Results

### Biogenesis of the membrane complex employs three *Acinetobacter*-specific envelope-associated proteins

**Fig 2:**
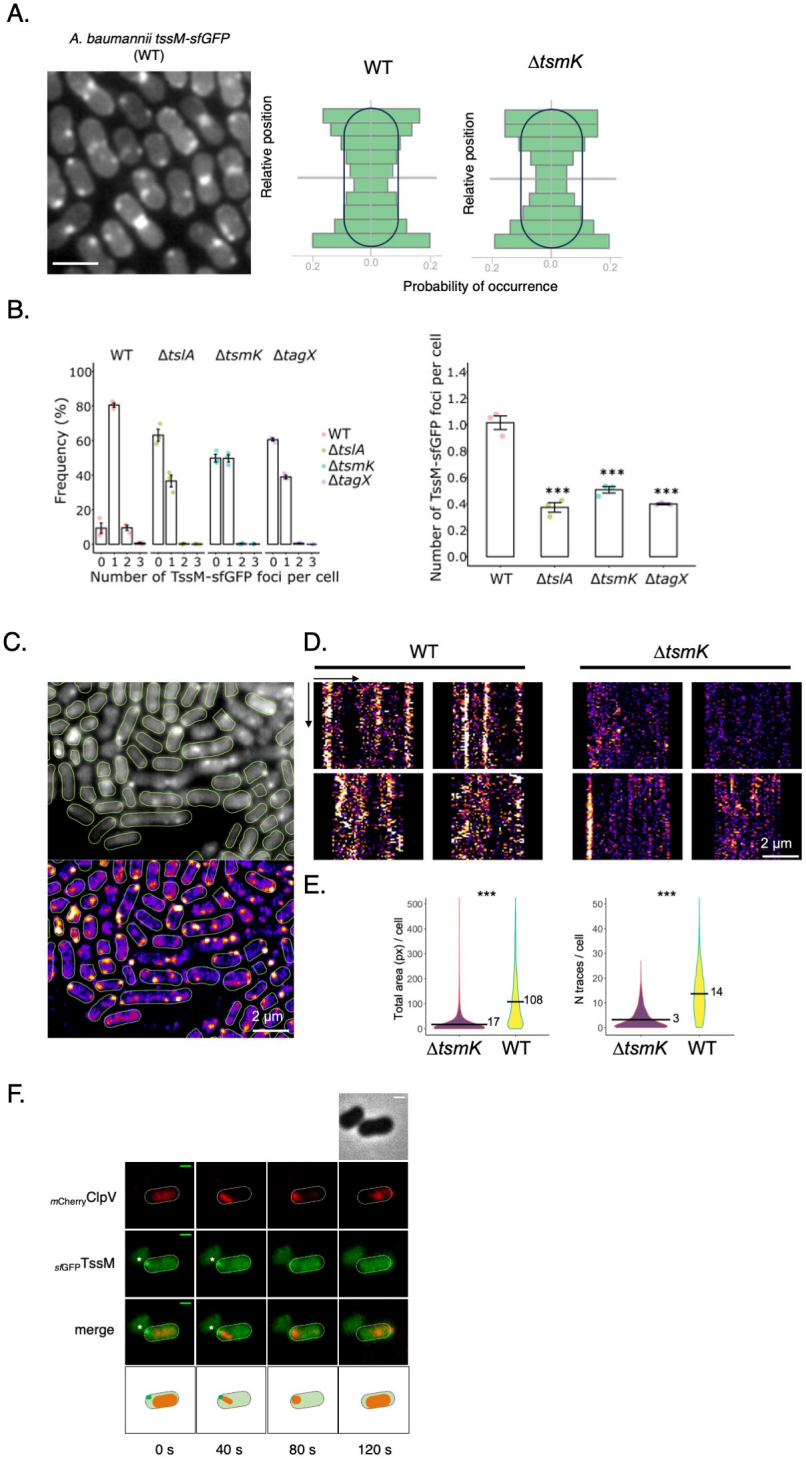
*A*. *baumannii* T6SS essential for the MC biogenesis. **(A)** Fluorescent cluster of the TssM^sfGFP^ protein in *A*. *baumannii tssM-sf*GFP strain. On the left panel, the images represent the average over a time lapse acquisition of 61 frames (10s / frame). scale = 2 μm. The right panel represents the histograms of cluster distribution of the main axis of the cell with a preferential accumulation at the poles of the cell. (n > 1000 cells, two biological replicates). Comparison of the cellular distribution of TssM-*sf*GFP foci between the WT and *tsmK* mutant (right panels). **(B)** Statistical analysis of the amount of fluorescence foci in the different mutants. On the left, a graphical representation of the percentage of cells with zero (n = 0), one (n = 1), two (n = 2), or more than three (n = 3) foci in the different *A*. *baumannii* mutants. On the right, a graphical representation of the average number of foci per cell in the different mutants. The experiment was performed in triplicate on three different groups of cells. **(C-E)** Dynamic study of the fluorescence foci of the TssM-*sf*GFP in WT and *tsmK* mutant. Fluorescence microscopy in TIRF mode captured an average of 61 images every 10 seconds, revealing multiple fluorescent foci within the cells. Cell masks were generated using the Cellpose cyto2 model, and FIJI’s MicrobeJ plugin, set in "rod-shape" mode. The lower panel shows the average of images processed with a bandpass FFT filter and background subtraction that enhances the foci contrast. **(D)** Kymographs derived from intensity profiles measured along the cell contour, representing time (61 images every 10 seconds) on the vertical axis and cell contour length in microns on the horizontal axis. Traces of foci movement along the cell contour are evident, with fixed and stable foci yielding vertical traces, some corresponding to the whole acquisition duration (the height of the kymograph = 610 seconds). **(E)**. Violin plots for the quantitative analysis of foci traces between the WT and *tsmK* mutant (3 biological replications). The difference in means was tested by t.test (R software) and yields a p value < 0.0001. (**F**) Biogenesis of the *A*. *baumannii*-T6SS MC and the original degradation of the tail after contraction. Time-lapse fluorescence microscopy recordings showing localization and dynamics of the ^*m*Cherry^ClpV and TssM^*sf*GFP^ fusions proteins. Individual images were taken every 40 sec. The positions of the foci are indicated by an asterisk. The scale bars are 1 μm. The lines (from top to bottom) represent the phase contrast, the *m*Cherry channel, the *sf*GFP channel and the superposition of the two channels. Below, a schematic representation of the sequential biogenesis of the T6SS membrane complex.

The presence of specific *A*. *baumannii* T6SS components and the absence of TssJ, prompted us to investigate their role in the functioning and assembly of the trans-envelope spanning complex. Bioinformatic analysis revealed five genes encode for membrane-associated proteins (Fig 1A, green stars). Four are *A*. *baumannii*-specific proteins and two of them (TslA/AsaA and TagX/AsaE) have already been characterized (see Introduction). The protein encoded by the A1S_1301 locus has an unknow function (Fig 1A, green stars), but bioinformatic analysis suggested a putative implication in phospholipid biogenesis due to its structural homology with β-ketoacyl synthase. We renamed the protein encoded by the A1S_1301 (*asaB)* gene TsmK (**T**ype **s**ix secretion **m**embrane complex assembly pseudo-**K**etoacyl synthase). To test the importance of this protein in T6SS functioning, we deleted the corresponding gene in the *A*. *baumannii* ATCC 17978 strain and performed phenotypic assessment of the T6SS activity. For comparison, we have also designed mutant strains that are deleted for genes previously reported to be essential to *A*. *baumannii* T6SS functioning. As previously described, deletion of *tagN* had no impact on T6SS functioning demonstrated by the T6SS-dependent secretion of the Hcp protein (Fig 1B) and the killing of a prey *E*. *coli* strain (Fig 1C), which were both at levels comparable with the parental *A*. *baumannii* ATCC 17978 strain. As observed by Ringel *et al*. [24], deletion of *tslA (asaA)* had an intermediate effect and did not abolish the Hcp secretion and the T6SS-dependent killing of *E*. *coli* (Figs 1B, 1C and S1A). Interestingly, the deletion of *tsmK* completely abolished T6SS functioning (Figs 1B, 1C and S1A) to the same extent as the deletion of *tssM* or *tagX* (*asaE*), both encoding essential components of the T6SS [7,19]. The defect in Hcp secretion of the *tsmK* deletion mutant was complemented to WT level by a plasmid with an inducible copy of the gene, indicating no polar effect of the deletion (S1B Fig). To investigate the involvement of the three proteins TslA, TsmK and TagX in the biogenesis of the *A*. *baumannii* T6SS MC, we needed to monitor its assembly using a visual and quantitative assay. The *A*. *baumannii* MC has never been studied nor observed directly. In order to monitor the assembly and dynamic of the MC, we generated different fluorescent chimera with conserved T6SS components (S2A and S2B Fig). Only the chromosomally expressed TssM^*sf*GFP^ was functional and able to assemble readily detectable foci using live fluorescence microscopy (Fig 2A). The fluorescent foci were detected as local maxima by MicrobeJ software and localized relatively to the medial axis of the cell. The position along the medial axis was normalized to [-1, +1] and the distribution presented as histograms (Fig 2A, right panel). Analysis of foci distribution showed that the *A*. *baumannii* MC was randomly distributed around the cell periphery with a preferential accumulation at the bacterial poles (n > 1000 cells, two biological replicates). Using a different fluorescent fusion (mCherry) did not alter the pattern of distribution of TssM (S2C Fig, left panel). As expected, mutations affecting conserved essential T6SS components of the tail (TssB-C or ClpV) did not alter this localization pattern (S2C Fig, right panel). To investigate the involvement of the three proteins TslA, TsmK and TagX in the biogenesis of the *A*. *baumannii* T6SS MC, we generated in-frame deletions of the different genes in the recombinant *A*. *baumannii* ATCC 17978::TssM^*sf*GFP^ strain. Live fluorescent recording and quantification of MC foci showed that the parental strain displayed an average of 1.0 foci per cell (1.01 ± 0.09) (Figs 2B and S2D). However, a severe loss of MC assembly was observed in the different mutants (Figs 2B and S2D). In the *tslA* and *tsmK* mutants we counted an average 0.4 (0.37 ± 0.06) and 0.5 foci (0.51 ± 0.04) per cell, respectively. Similarly, in the *tagX* mutant we observed an average of 0.4 (0.40 ± 0.01) foci per cell. Overall, the decreased number of average foci per cell in the mutants can be interpreted by an impaired stability of the MC. Control experiments showed that TssM production was identical in all the various mutants (S2E Fig), and that their deletion did not trigger any polar effects on the expression of *tssM*. In conclusion, TslA, TagX, and the uncharacterized TsmK proteins are required for both functioning of the T6SS in *A*. *baumannii* and for the assembly of the trans-envelope MC.

Since TsmK is a newly described T6SS component, we further characterized its role in MC biogenesis. Even though the *tsmK* mutant exhibited decreased number of TssM^*sf*GFP^ foci, their overall cellular distribution remained unchanged (Fig 2A). Interestingly, the dynamic pattern of MC assembly is dramatically altered in the *tsmK* mutant. We recorded traces of foci movement along the cell contour, with fixed and stable foci yielding vertical traces, some corresponding to the whole acquisition duration (610 seconds) (Fig 2C and 2D). A quantitative analysis of foci traces revealed significant differences between the WT and *tsmK* mutant: WT exhibited larger traces (108 pixels, sd = 113) compared to the mutant (17.2 px, sd = 48.7), along with a higher number of traces per cell (13.7, sd = 8.7) versus (3.22, sd = 3.9) in the *tsmK* mutant (*WT n = 2850*, *tsmK n = 3189*, *for 3 biological replications*) (Fig 2E). We thus concluded that TsmK is important for the stability and dynamic of the TssM-containing membrane complex (MC).

The MC is preferentially, but not exclusively localized to the cell pole. We wondered if other T6SS components are enriched at this specific location. Fluorescent labelling of sheath subunits has been often used in other bacteria to monitor T6SS dynamic. However, it is not possible to genetically engineer TssB^*sf*GFP^ fusion at the chromosomal T6SS locus in *A*. *baumannii* due to the translational coupling of both *tssB* and *tssC* genes. However, the dynamic of the recycling ATPase ClpV was previously reported as a proxy to monitor the sheath dynamic, i.e. assembly, contraction and disassembly [5,25]. We constructed the dually labelled strain *A*. *baumannii*::TssM^*sf*GFP^::^*m*Cherry^ClpV and observed single cell behavior using live fluorescence microscopy. TssM^*sf*GFP^ forms static foci that are unstable over time, appearing and disappearing during the observation. The ^*m*Cherry^ClpV signal is highly evolutive and dynamic, forming diffuse signal first (panel 0s), followed by long rod-shaped signal (panel 40s), then short rod-shaped signal (panel 80s) and ultimately evolves as diffuse signal (panel 120s) (Figs 2F and S2F). We concluded that ClpV follows the same behavior as the T6SS sheath. The ^*m*Cherry^ClpV signal (long rod-shaped) can be observed colocalized with that of TssM^*sf*GFP^. We thus conclude that the polar MC foci are used for TTC dynamic assembly, consequently they are functional spots for the *A*. *baumannii* T6SS. Interestingly, after contraction of the sheath, when ^*m*Cherry^ClpV behaves as short rod-shaped or diffuse signals, the TssM^*sf*GFP^ signal completely disappears. These results suggest that upon contraction of the sheath, the *A*. *baumannii* MC disassembles. We concluded that the *A*. *baumannii* T6SS MC undergoes a cycle of assembly and disassembly coordinated with the sheath dynamic.

### An intricate interaction network assembles the *A*. *baumannii* membrane super-complex

**Fig 3:**
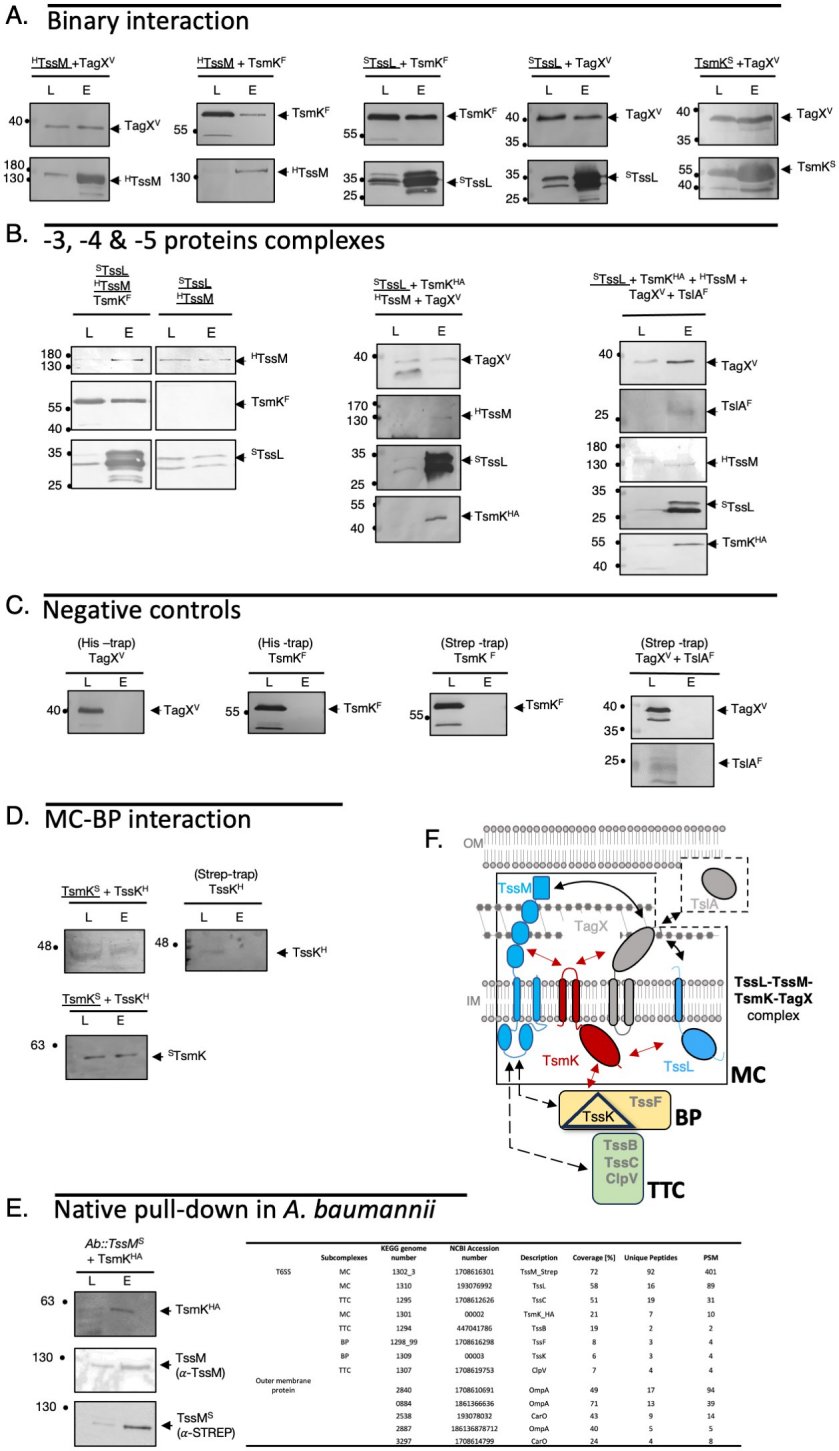
Determination of the protein-protein interaction network (PPIN) of *A*. *baumannii* T6SS membrane complex. **(A)** Western blot probing binary interaction between TagX, TssM, TssL and TsmK respectively. **(B)** Co-purification of the 3-, 4- and 5-protein complexes. **(A-D)** Detergent-solubilized extract of *E*. *coli BL21*(DE3) cells producing the indicated protein were submitted to an affinity purification step on Strep- or His- Trap. The detergent-solubilized total membrane (L), and eluate (E) were subjected to denaturing 12.5%-polyacrylamide gel electrophoresis (PAGE) and immunodetected with the appropriate antibody. The composition of the protein mix is indicated on the top of each panel, the protein that binds to the column is underlined. Immunodetected proteins are indicated on the right. Molecular weight markers (in kDa) are indicated on the left. Tags: H, 6×His; S, Strep-tag; F, FLAG; HA, Hemagglutinin; V, VSVG. **(C)** Negative controls. **(D)** Western blot probing the binary interaction between TsmK and TssK (left panel), including negative control (TssK alone, right panel). **(E)** Native pull-down of TssM from A. baumannii cells. Western blot probing the identified interaction partners (left panel). Mass spectrometry analysis of TssM copurified interactants (right panel).**(F)** Schematic interaction network determined by co-purification and topology of the *A*. *baumannii* T6SS-MC full-length proteins. Interactions between proteins are represented with arrows: TsmK is in the center of the interaction network (red arrows). Indirect interactions (native pull-down are shown by dashed double-head arrows.

We sought to determine how the canonical TssL-TssM T6SS MC components connect with the *A*. *baumannii* T6SS-specific proteins. We have thus determined the protein-protein interaction network (PPIN) by co-purification experiments using full-length membrane proteins in detergent micelles from heterologous production in *E*. *coli* (see Material & methods). Bioinformatic analysis identified two transmembrane helices in TsmK that delineates a possible large cytoplasmic domain (S3A Fig), accounting for 87% of the full-length protein. The *tssL*, *tssM*, *tsmK*, *tagX* and *tslA* genes were co-expressed in *E*. *coli BL21*(DE3). Constructs were designed to add StrepII, 8His, VSVG and Flag tags at the amino N-terminus of TssL (^S^TssL, 30.7 kDa), N-terminus of TssM (^H^TssM, 138.7 kDa), carboxy C-terminus of TsmK (TsmK^F^, 54.8 kDa), C-terminus of TagX (TagX^V^, 24.5 kDa) and C-terminus of TslA (TslA^F^, 25.4 kDa), respectively (S1 Table). Total membrane fractions were isolated and solubilized using detergents. Affinity chromatography was performed on either Strep- or His- Trap-HP column. By interacting binarily with TssM, TssL and TagX, the newly identified TsmK appeared to occupy a central position in MC PPIN (Fig 3A and 3B). We also demonstrated that TagX interacts with TssM. In addition, by pulling down on TssL^S^ we isolated a protein complex composed of the three proteins TssL, TssM, and TsmK (Fig 3B). We further demonstrated that TagX and TslA were recruited to the complex, producing a large four and five-proteins membrane super-complex, respectively. When comparing the purification of TssL-TssM and TssL-TssM-TsmK complexes, we observed that the level of TssL and TssM were increased in the presence of TsmK (Fig 3B). All the tested proteins (except the Strep- or His-tagged baits) do not interact nonspecifically with the affinity resins (Fig 3C). We concluded that TsmK stabilizes the complex. We thus demonstrated an intricate PPIN connecting the 4 transmembrane proteins and TslA, revealing the original composition of the *A*. *baumannii* transenvelope T6SS super MC. By interacting simultaneously with TssL, TssM and TagX, the protein TsmK appeared to be a cornerstone in the *A*. *baumannii* MC. TslA and TagX can further interact with this core-complex, leading to the assembly of an envelope-spanning complex in *A*. *baumannii* T6SS.

We then asked if the large and bulky cytoplasmic domain of TsmK could contact the baseplate (BP). To answer to this question we co-produced in *E*. *coli* both TsmK and TssK. Pulling down experiment using TsmK^STREP^ as a bait allowed the co-purification of TssK (Fig 3D). No TssK was retained on the STREP column in the absence of TsmK. Consequently, TsmK is able to directly interact with TssK, a central component of the BP.

To validate the PPIN observed in *E*. *coli*, we sought to confirm these results using native pull-down directly in *A*. *baumannii*. For this purpose, *A*. *baumannii* ATCC 17978 was genetically modified to introduce an in-frame DNA sequence encoding a STREPII tag to TssM-Cter (see Material & methods). The *A*. *baumannii*::TssM^STREP^ retains a fully functional T6SS, as evaluated by Hcp secretion. In addition, to confirm the TssM-TsmK interaction observed in *E*. *coli*, we transform the *A*. *baumannii*::TssM^STREP^ strain with the plasmid pVRL2-TsmK^HA^. The detection of TsmK^HA^ is rendered possible by anti-HA western blot. Remarkably, TsmK co-purifies with TssM directly in *A*. *baumannii* (Fig 3E, left panel). We then analyzed the purified sample using mass spectrometry–based proteomics (see Material & methods). We further demonstrated that T6SS proteins belonging to the three functional sub-complexes are also co-purified by TssM in *A*. *baumannii*: TssL and TsmK (membrane complex, MC); TssK and TssF (baseplate, BP); TssB, TssC and ClpV (tail-tube complex, TTC) (Fig 3E, right panel). Together, these results enforce the presence of an entangled protein protein interaction network that build the T6SS nanomachine, which was already beginning to be unraveled with our reconstituted approach in *E*. *coli* (Fig 3F).

Interestingly, several outer membrane proteins were additionally copurified with TssM, of which OmpA (Fig 3E). Even though they could represent contaminants, however OmpA was already proposed to ensure the correct membrane localization of the T6SS in *A*. *baumannii* [23]. Our result suggests that the function of OmpA could be mediated by its interaction with TssM.

### Analysis of the *Acinetobacter*-specific T6SS protein TsmK

**Fig 4:**
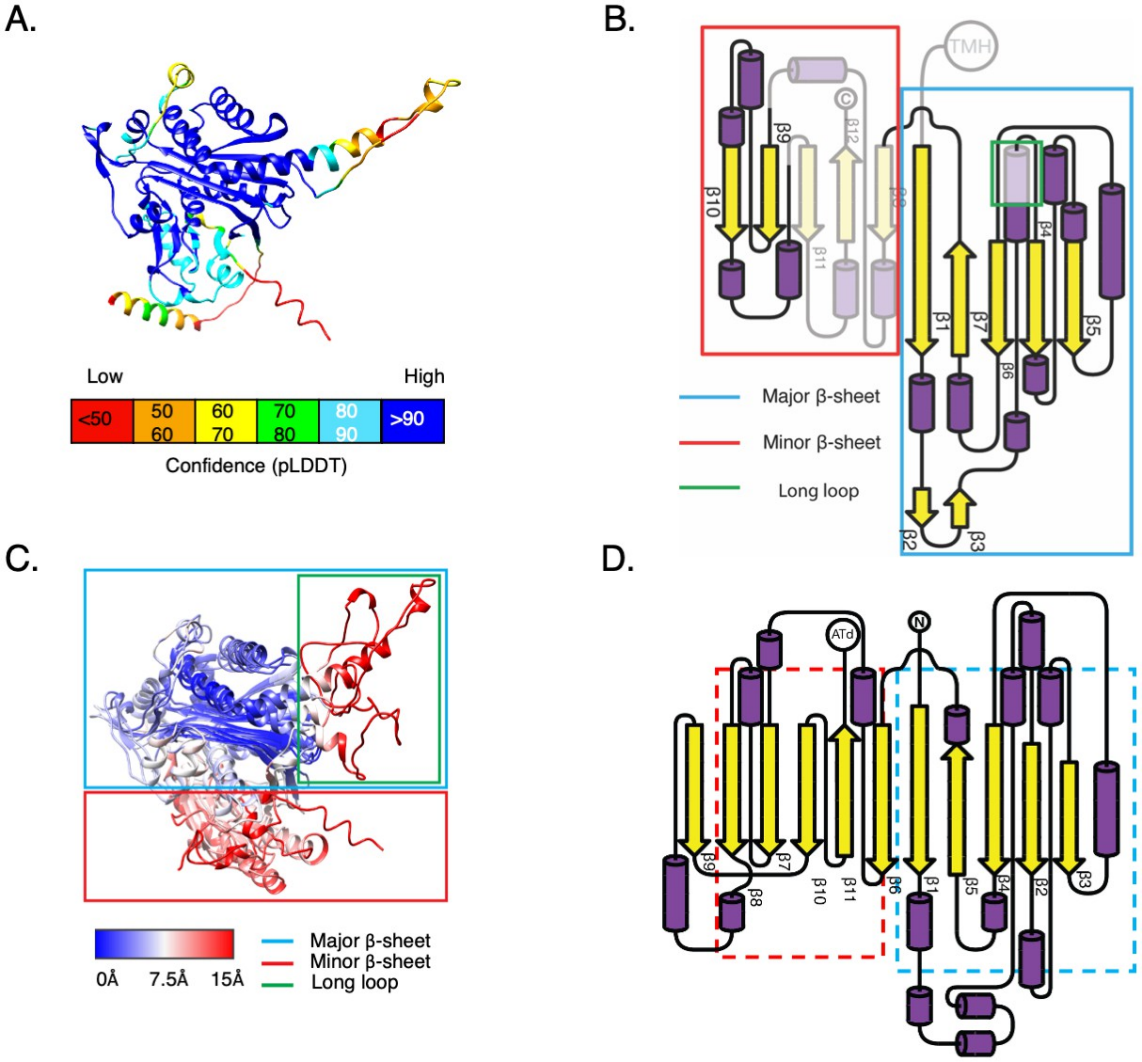
Structural prediction study of TsmK an *Acinetobacter*-specific protein related to Ketoacyl synthases. Analysis of the TsmK predicted structure. **(A)** AlphaFold2 confident score (predicted LDDT per position) mapped on the model. **(B)** Topology of the secondary elements of the predicted model for TsmK. The diagram depicts the secondary structure organization of the *A*. *baumannii* TsmK model generated with AlphaFold2. The major and minor β-sheets, as well as the long loop, are highlighted with colored squares. Inconsistent regions are represented with pale colors. Five antiparallel β-strands, β1, β7, β6, β4, and β5 assemble into a β-sheet (referred to as the major β-sheet). The α-helices are present at the loops formed between each pair of consecutive β-strand and decorate both sides of the β-sheet. AlphaFold2 predicted a second β-sheet (referred to as minor β-sheet) not present in TrRosetta and RaptorX predictions. This β-sheet is formed with the two β-strands of the converging hairpin (β9 and β10) and the β11, β12, and β8 **(C)** Structural superimposition of the three structural models of *A*. *baumannii* TsmK. The major and minor β-sheets as well as the long loop are highlighted with colored squares. Structural precision ranges between 0 and 15 Å (blue to red) **(D)** Topology of the secondary elements of the Ketoacyl synthase domain from Acyltransferase type I polyketide synthase (PKS) (PDB 4TKT). The homologous regions between TsmK and the KS domain of the polyketide synthase are highlighted in dashed colors.

Since TsmK plays a key role in the assembly of the *A*. *baumannii* T6SS MC, we sought to investigate its conservation and structural features. We conducted a sequence analysis to examine how the TsmK conservation level compares with the conservation level of TssM, TssB, and TssK in *Acinetobacter* and in *A*. *baumannii* only (S4A Fig). We observed that TsmK had a conservation level lower than that obtained for TssK, TssB, and TssM, thus suggesting a higher sequence heterogeneity (or alternatively sequence divergence) than that associated with other T6SS sequences. To study the presence of TsmK in *Acinetobacter* and other proteobacterial species, we performed a search of homologous sequences using BLASTp. We obtained 116 hits with a unique taxonomy ID (TaxID) and genome. 70% (81/116) of these hits belong to the genus *Acinetobacter*. Most of these sequences had a blast e-value below 1e-20 and covered at least 60% of the query sequence (S4B Fig). In S4B Fig, the red vertical and horizontal dotted lines represent the e-value (1e-20) and coverage thresholds (0.6), respectively, and are used to determine if a homolog is a TsmK sequence. On the other hand, the *non-Acinetobacter* hits (30%) had an e-value much higher than the threshold (1e-20) (most of them have an e-value greater than 1e-05 and a below 60% of coverage). This clear separation allowed us to classify the hits in true TsmK sequences found in *Acinetobacter* and similar sequences found elsewhere. We studied the co-occurrence of TsmK, TssM and TssJ using a two-step approach (S4C and S4D Fig). First, we analyzed *Acinetobacter* genomes containing the TsmK sequences. 96% of them contained a TssM gene, while none of them had a TssJ gene. TssJ and TssM were found in 77.1% and 97.1% of the *non-Acinetobacter* genomes, respectively (see Material and methods). Second, we searched for TsmK, TssM, and TssJ genes in the 1350 *Acinetobacter* genomes available, using TssB as an indicator of a complete T6SS operon. None of the complete 893 operons had TssJ, 90.5% contained TssM, and 90.0% contained TsmK. Importantly, 82.2% had TssM and TsmK simultaneously (S4D Fig). These two observations let us conclude that co-occurrence of TsmK and TssM, and the simultaneous absence of TssJ in *Acinetobacter* are statistically robust properties of *A*. *baumannii* T6SS. To investigate the function of TsmK, we computed structural models based on its sequence using three *ab initio* predictors AlphaFold2 [58], TrRosetta [60] and RaptorX [26]. All three models essentially agree on the global fold and arrangement of the core of TsmK (Figs 4A, 4B and S4E). The three models disagree in some parts, referred to as the inconsistent regions (Figs 4B, S4E and S4F). Essentially the inconsistent regions are: 1) the two N-terminal transmembrane helices 2) a long loop between β1 and β4, 3) the minor β-sheet, with the exclusion of β9-β10 hairpin with associated helices (Fig 4B). The inconsistent regions have a structural precision that ranges between 7 and 15 Å, and a low AlphaFold2 confidence score (Fig 4C). On the contrary, the consistent regions are the major β-sheet together with β9-β10 hairpin, with all the associated helical decoration (Fig 4C). The consistent regions have a structural precision that ranges between 0 and 7 Å, and a high AlphaFold2 confidence score (Fig 4C). Using the DALI server [27], we queried the structure of the consistent region of TsmK to find the closest structural homolog. The protein with the highest structural similarity was the Ketoacyl Synthase (KS) domain from Acyltransferase type I polyketide synthase (PKS) (PDB 4TKT, [28]) from *Streptomyces platensis* (Figs 4D and S4G). TsmK and the KS domain shared a low sequence identity (10%) (S4H Fig). The structural alignment of the KS domain with the TsmK model revealed how the major and minor β-sheets fit the corresponding parts of the KS domain. The resulting structure-based sequence alignment showed how the TsmK model lacks two of the three conserved catalytic residues involved in the fatty acid modification (S4H Fig). A structural inspection of the conserved catalytic residue His 366 found in the TsmK model did not reveal any cysteine in the surroundings that could substitute the catalytic cysteine of PKS. In fact, the unique cysteine found on TsmK was located in β7, far from the hypothetical conserved catalytic site. Taken all together, these results suggest that TsmK had retained structural features of the fatty-acid modifying enzyme, but not its function. One of the functions of TssJ in the T6SS is to stabilize the MC through its interaction with the central pilar of the trans-envelope complex, the protein TssM [19,29]. We thus demonstrated here that TsmK could have been co-opted by the *A*. *baumannii* T6SS to fulfill such a stabilizing function.

### *A*. *baumannii* TssM undergoes a specific dynamic in the cell envelope

**Fig 5:**
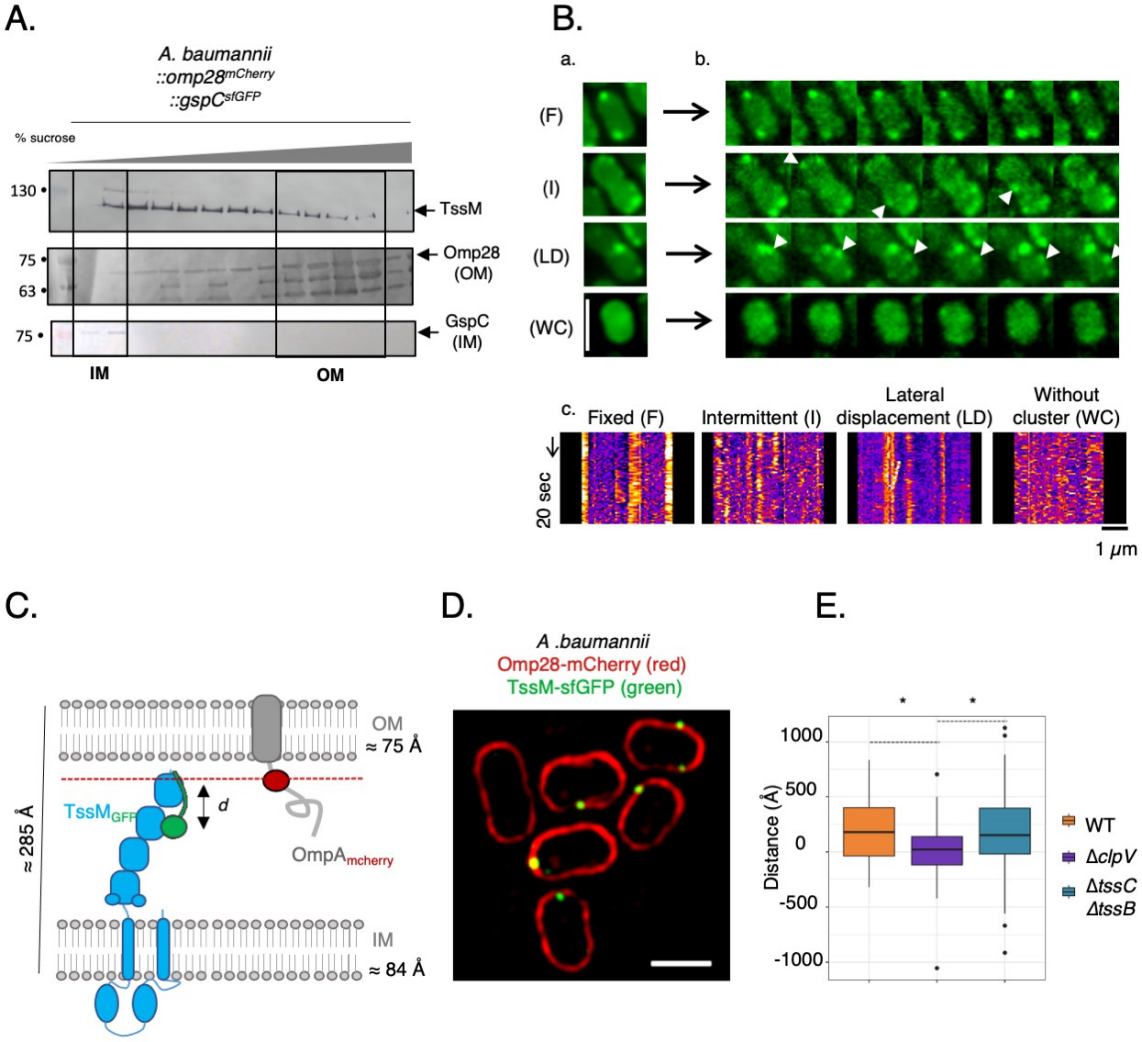
Characterization of TssM location. **(A)** TssM location determined by sucrose gradient. *A*. *baumannii* ATCC 17978 strains were grown, and the outer (OM) and inner (IM) membranes were separated by a continuous sucrose gradient (35%-60%). After centrifugation, 750ul samples were taken from the top to the bottom of the gradient. The samples were subjected to denaturing 12.5%- polyacrylamide gel electrophoresis (PAGE) and immunodetected with the appropriate antibody (including synthetics polyclonal TssM antibody). Immunodetected proteins are indicated on the right. Molecular weight markers (in kDa) are indicated in the left. Tags: GspC^*sf*GFP^ (T2SS IM protein), Omp28^*m*Cherry^ (OM porin). **(B)** Dynamics of fluorescent clusters of the TssM protein. Cells were recorded in TIRF illumination during 10 min each 10 seconds **(a)**. The column in panel **(a)** shows the corresponding average image of the 4 cells. Panel **(b)** shows 6 time points of these 4 cells. The arrows point to the cluster fluctuations (line 2) or their lateral displacement (line 3). The lower panels **(c)** are kymographs that illustrate 4 types of movements: fixed clusters (F), intermittent clusters (I), lateral displacement (L), cell without clusters (WC). The kymographs were constructed from the intensity profile of the cell contour. Scale: 2 μm **(C)** In *A*. *baumannii* ATCC 17978, the fusion of sfGFP to TssM C-terminal (TssM-sfGFP) and mCherry to a majority porin (Omp28-mCherry) was used to observe the location of the TssM C-terminal. The porin was used as a proxy to label the outer membrane. **(D)** Structured illumination microscopy (SIM) images of the TssM^sfGFP^ regarding the Omp28^mCherry^ label. The scale bar is 1μm. **(E)** Box plot representing the distances measured between the TssM foci and the mCherry label in a WT, a *clpV* mutant and a *tssB-tssC* mutant. The difference in position is equivalent to the distance between the two observed fluorophores and was measured in n = 60 bacteria (WT_ *ΔclpV*: t 3.1331, df = 105.39, p-value = 0.00224; WT_ *ΔtssBΔtssC*: t = 0.024481, df = 100.92, p-value = 0.9805; *ΔclpV*_Δ*tssBΔtssC*: t = -2.6278, df = 95.461, p-value = 0.01002).

The second function of TssJ is to anchor the MC into the outer membrane, allowing TssM sub-domains to cross the lipid bilayer [19]. The absence of a TssJ-like component in *A*. *baumannii* prompted us to investigate the specific sub-cellular location and behavior of TssM. We first determined the membrane localization of TssM using sucrose gradient fractionation of *A*. *baumannii* total membranes (Mat. & Methods). TssM appeared strongly associated with both the inner membrane and with the Omp28-containing fraction (Fig 5A). Consequently, even in the absence of a TssJ homolog TssM is still anchored in the outer membrane fraction. To further characterize the location of TssM we have monitored its stability over time using fluorescence microscopy observation of the GFP fusion protein. The time lapse stack was reduced in one image by the average projection. The region of the image in S5A Fig illustrates that almost all cells harbored one or more stable clusters over time that are clearly visible in the average image (S5A Fig). However, observation of the recording also shows very dynamic clusters that move or appear and disappear rapidly and some cells where clusters are not well visible (S1 Movie, Fig 5B sub-panels a-b). To characterize these behaviors, we plotted the kymographs corresponding to the intensity profile of the cell contour. We could record four types of movements: fixed clusters (F), intermittent clusters (I), lateral displacement (LD), cell without clusters (WC) (Fig 5B, sub-panels c), illustrated by representative average images (Fig 5B, sub-panels a-b). The different positions and movements of TssM foci are reminiscent to the TagB localization and dynamic in the *P*. *putida* T6SS [18]. Indeed, TagB forms transient foci which accumulate and abruptly disappear over time and then re-appear in the same or neighboring cells. However, TagB is a cytoplasmic protein that is recruited at the baseplate where it stabilizes sheath polymerization. Once the sheath contracts, TagB foci are dispersed. The TssM dynamic can be better understand in the context of its colocalization with the ClpV ATPase (Fig 2G). In conclusion, we propose that TssM foci assemble and then disappear after sheath contraction, which could then explain this intermittent appearance.

The heterogeneity in sub-cellular localization and dynamic of the *A*. *baumannii* MC invited us to look at its behavior relative to the cell envelope *in vivo*. For this purpose, we performed super-resolution 3D structured illumination microscopy (SIM) on intact *A*. *baumannii* cells. The Omp28^mCherry^ porin was used as a proxy to label the outer membrane and TssM location was monitored by following the *sf*GFP signal, located at its C-terminus (Fig 5C). TssM foci appeared as bright green spots co-localized near the diffuse and linear outer membrane signal of Omp28 (Fig 5D). Assuming that the TssM fluorescent signal is produced by a single particle we can estimate its localization with sub-pixel accuracy by fitting a Gaussian curve to the TssM fluorescent signal along a transversal intensity profile (S5B and S5C Fig). The estimated position of TssM corresponds to the peak of the fitted curve. With the same approach we can estimate the likely position of the OM signal along the same intensity profile (S5C Fig). With these two estimated positions we can relatively localize the two signals for TssM and OM. Wildtype cells (WT) exhibit a relative position slightly shifted inward, compared with a random localization centered at 0 shift. In the absence of ClpV (*ΔclpV*) TssM showed a localization centered around the Omp28 signal (Fig 5D and 5E) as expected for a random localization inward/outward. Conversely, the localization of TssM without TssBC (*ΔtssC*, *ΔtssB*) also presented a slightly inward-shifted signal. In the absence of dynamic recordings and within the limits of SIM microscopy resolution, speculatively this result could mean that ClpV activity is required to maintain the inward positional shift of TssM in the OM.

### A C-terminal glycine-serine linker specific to *Acinetobacter* TssM is crucial for T6SS functioning

**Fig 6:**
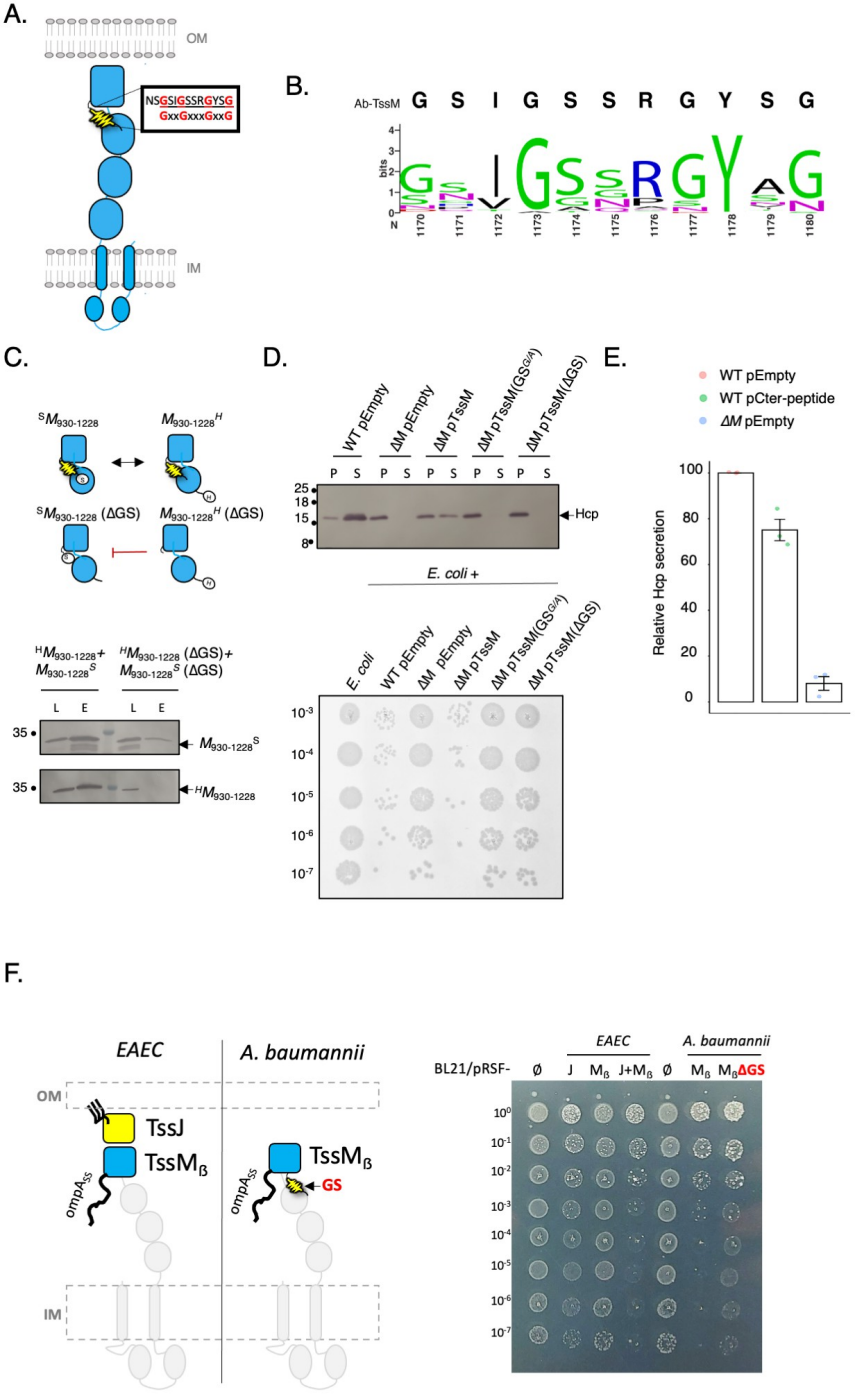
The *A*. *baumannii-*specific TssM C-terminal GS-linker is essential for T6SS assembly and functioning. **(A)** Schematic topology of *A*. *baumannii* TssM protein. This protein has a huge periplasmic domain composed of three alpha domains (circular shape) and one beta domain (scare shape) on the C-terminal. On the C-terminal, the protein has a GxxGxxxGxxG helix (the “GS-linker”, in yellow) inserted between the last α- and the β- domain of the protein. **(B)** The 807 aligned TssM sequences were extracted from the set of *Acinetobacter* genomes with a complete T6SS operon. The height of each letter represents the information content of the corresponding residue at a given position in bits. *A*. *baumannii* strain ATCC 17978 TssM (on top of the sequence logo) sequence was used as a reference position. **(C)** TssM C-terminal oligomerization depends on the “GS-linker” motif. On the top, a schematic representation of the TssM C- terminal constructions used (the periplasmic domain between 930 and 1228 amino acids) to determine the interactions. Soluble extracts of *E*. *coli* BL21(DE3) cells producing the indicated protein were submitted to a simple affinity purification step on Strep-Trap. The lysate (total soluble extracts, L), and eluate (E) were subjected to denaturing 12.5%-polyacrylamide gel electrophoresis (PAGE) and immunodetected with the appropriate antibody. Immunodetected proteins are indicated on the right. Molecular weight markers (in kDa) are indicated in the left. Tags: H, 6×His; S, Strep-tag. **(D)** Upper panel, western blot assays probing for Hcp secretion in whole-cell pellets (P) and supernatants (S), by *A*. *baumannii* ATCC 17978 wildtype with the plasmid control, the *ΔtssM* mutant and the complemented mutant strain. The last two show the phenotype of *ΔtssM* mutant owning a plasmid pVRL2- with tssM mutated (Gly→Ala substitution in position 1172, 1175 and 1179 or a deletion of the alpha helix between the 1172 and 1179 residues). The TssM expression was induced by adding 0.1% arabinose. Lower panel, bacterial competition experiments. Survival of *E*. *coli* rifampicin-resistant after incubation with ATCC 17978 wildtype with the plasmid control, the *ΔtssM* mutant and the complemented mutant strain. **(E)** The “Small Domain Interference” (SDI) validate the importance of TssM-CTD. Dot blot quantification of Hcp secretion in wildtype with the plasmid control (pVRL2-) or a plasmid overexpressing the TssM C-terminal peptide, and in the *ΔtssM* mutant with a plasmid control. The C-terminal peptide was induced by adding 1% arabinose. **(F)** Outer membrane permeability test. Scheme representing the different protein constructs produced in *E*. *coli* BL21 (left panel). Exponential-phase cultures of *E*. *coli* overproducing different protein domains were adjusted for OD600 and serial diluted onto LB media supplemented with vancomycin 10μg/mL and 0.05mM IPTG (right panel).

We then sought to identify molecular features that could render TssM independent of TssJ for its anchoring to the outer membrane. In EAEC, TssM interacts with TssJ through the C-terminal domain (CTD), notably the β-strand domain (S6A Fig) [29]. We thus focused our attention on the conservation of the CTD. Interestingly, the interaction region between TssJ and TssM is well conserved between *A*. *baumannii* and other proteobacterial species (S6A Fig). The multiple sequence analysis of *Acinetobacter* TssM sequences highlighted the presence of a conserved GxxGxxxGxxG motif in the TssM CTD, which is not observed in other proteobacterial species (Figs 6A, 6B, S6A and S6B). From the amino-acid composition of the motif, rich in glycine and serine residues, and its secondary structure prediction, we hypothesized that this peptide is a linker or a random-coil region. We have named this motif GS-linker in reference to a “Glycine/Serine-rich linker”. We have modelled the structure of *A*. *baumannii* using Alphafold2 (AF2) (S6C Fig). Interestingly, this GS-linker has a loop-like conformation which is located at the interface between the last alpha α- and β-domains, at the same position occupied by a short hydrophobic 3(10)-helix for EAEC (S6C Fig sub-panel a-b). To understand the contribution of the GS-linker to TssM-TssM interaction, we monitored the homotypic interaction of part of the TssM periplasmic domain (TssM_930-1228_) (Fig 6C, upper panel). A pull-down assay between Strep- and His-tagged versions of TssM revealed that TssM_930-1228_ domain is able to homo-multimerize (Fig 6C, lower panel). On the contrary, deletion of GS-linker in TssM_930-1228_ blocks this interaction, suggesting that GS-linker is essential for the multimerization of TssM periplasmic domain. To assess the implication of the GS-linker in the functioning of the *A*. *baumannii* T6SS, we designed by targeting mutagenesis (i) a TssM mutated protein where the first three Gly were replaced by three Ala residues and (ii) a TssM mutant where the GS-linker was completely deleted (see Material & methods). We investigated the functionality of these TssM variants to restore the Hcp secretion phenotype in the *tssM* deleted strain by trans-complementation. Expression of the WT *tssM* allele was able to complement Hcp secretion of a *tssM* mutant (Fig 6D, upper panel). However, complementation by a “*GS*^*G/A*^” or “*ΔGS*” mutated alleles failed to restore the T6SS phenotype in *A*. *baumannii*. We checked that the mutations did not affect the level of TssM production, which was equivalent to WT TssM (S6D Fig.). The impact of the mutations of the GS-linker on the functionality of the *A*. *baumannii* T6SS was further validated by competition assay (Fig 6D, bottom panel). Contrary the WT *tssM* allele, the mutated alleles (ΔGS, G/A) both failed to restore killing of the *E*. *coli* prey.

Interestingly, the AF2 models of TssM-CTD with ΔGS display a displacement of the β-strand domain with respect to native position observed in the EAEC and WT structures (S6C Fig sub-panel c-d). To complement our knock-out approach, we performed “Small Domain Interference” (SDI) to validate the importance of TssM-CTD. We have recently used SDI or ectopic expression of peptide to surgically dissect the assembly of the T6SS wedge complex [30,31]. If the TssM-CTD is important for the multimerization of the MC and for OM anchoring, then ectopic production of this peptide outside the context of the full length TssM should interfere through competition with the functioning of the T6SS. Indeed, periplasmic overproduction of the TssM C-terminal peptide alone, encompassing the GS-linker reduced significantly (25% decrease) the Hcp secretion in the WT *A*. *baumannii* (Fig 6E). Altogether, these results indicated that the TssM GS-linker and its glycine composition is crucial for the functioning of the *A*. *baumannii* T6SS.

We then pursued to understand the involvement of TssM_ẞ_ domain in the crossing of the outer membrane and the specific role played by the GS-linker. We reasoned that anchoring of the TssM_ẞ_ domain in the outer membrane would increase the permeability of the outer membrane toward toxic compounds, a phenomenon previously observed with membrane-spanning proteins [32]. For that, we compared the permeability of the outer membrane of *E*. *coli* producing different periplasmic constructs in the presence of vancomycin, a bulky antibiotic that is normally incapable of crossing the outer membrane of Gram-negative bacteria. Two constructs were designed from the *EAEC* MC (TssJ, TssM_ẞ_ domain) and two constructs from the *A*. *baumannii* MC (TssM_ẞ_ domain, TssM_ẞ_ + ΔGS-linker) (Fig 6F, left panel). Of note, TssM_ẞ_ constructs are produced into the *E*. *coli* periplasm owing to a translational fusion with the N-terminal OmpA signal sequence. Overproduction of both TssJ and the TssM_ẞ_ domain of *EAEC* are both needed to trigger vancomycin sensitivity to *E*. *coli*, but not the individual proteins (Fig 6F, right panel). Interestingly, the *A*. *baumannii* TssM_ẞ_ domain alone rendered *E*. *coli* vulnerable to vancomycin. Conversely, deletion of the GS-linker abolished the outer membrane permeability. Together, these results suggest that the *A*. *baumannii* MC is anchor to and crosses the OM using the TssM_ẞ_ domain and that the GS-linker plays a pivotal role in this process.

We have identified two players that compensate the absence of TssJ functions in *A*. *baumannii* T6SS: the protein TsmK for the stabilization of the MC and the C-terminal GS-linker of TssM to help the oligomerization of TssM. To confirm this evolutionary scenario, we used a bio-informatic approach to study the co-occurrence of the three molecular events: the TssM GS-linker, the presence of TsmK, and the absence of TssJ in *Acinetobacter*. We analyzed the co-occurrence of these three conditions in the set of 807 *Acinetobacter* genomes containing both a TssM and TssB gene. The predominant configuration was the co-occurrence of the TssM GS-linker with the TsmK in the absence of TssJ, which was found in 88.4% and 82.8% of *Acinetobacter baumannii* and *non-baumannii* genomes, respectively. As a control, we also analyzed a set of 1096 non*-Acinetobacter* genomes containing both a tssM and a tssB gene (Fig 6F). Here, the predominant configuration was the lack of the TssM GS-linker motif with the absence of TsmK and the presence of TssJ, which was found in 61.5% of the genomes. The second most common configuration was the lack of the TssM GS-linker motif with the absence of TsmK and TssJ, which is found in 27.5% of the genomes. We have thus established a connection between the presence of both TsmK and TssM, the TssM GS-linker and the absence of TssJ as a key evolutionary event that shaped the *A*. *baumannii T6SS*.

## Discussion

The T6SS proteins assemble into a macromolecular apparatus across the bacterial cell envelope, dedicated to the delivery of a wide range of effectors, targeting prokaryotes or eukaryotes [3,33]. Most of the T6SS core components are conserved across bacterial species and have been the subject of in-depth functional and structural studies [20,33,34]. However, there are a number of species-specific T6SS proteins that have been discovered using bioinformatic tools and, in rare cases, experimentally characterized for their specific functions. These proteins are either key structural elements of the nanomachine or regulator of the T6SS assembly and functioning [7,35–37]. Studying these accessory components is of paramount importance to spot particular features of certain T6SS and to understand their adaptation to the microbe’s environment. The goal of this study was to characterize the composition, the structural organization and functioning of the *A*. *baumannii* T6SS membrane complex (MC).

Firstly, we observed that the functioning of the *A*. *baumannii* T6SS requires the uncharacterized protein TsmK, encoded by the gene *A1S_1301*. This protein, conserved in *Acinetobacter*, participates in the formation of an unorthodox T6SS core complex. Moreover, our bioinformatic analysis suggests that TsmK has retained the fold but not the catalytic properties of β-ketoacyl synthases. This observation is an excellent illustration of molecular tinkering, an evolutionary process that transforms pre-existing genes or parts of genes to produce new roles or biological functions, for a novel adaptive purpose. Likewise, the T6SS apparatus has been built upon the co-option of contractile tail phage genes assembled with bacterial genes encoding transmembrane proteins from other cellular machines [38]. β-ketoacyl synthases are multifunctional fatty acid synthases involved in the bacterial fatty acid pathway that is essential for membrane synthesis and a range of other metabolic and cellular functions. The co-option of such a protein raises questions about the specific function of TsmK in the T6SS, other than the stabilization of TssM and TssL complex. Recently, a house-keeping lytic transglycosilase MltE was demonstrated to have been co-opted by the EAEC T6SS to guarantee the proper assembly of the T6SS MC in the bacterial cell envelope [36]. As we delve deeper into the examination of species-specific T6SS components, we will uncover a broader array of molecular adaptations that have played a pivotal role in shaping the T6SS throughout its evolutionary journey in distinct environments. We can thus propose that TsmK is an *Acinetobacter*-specific T6SS component that could have been re-routed from its initial function in fatty acid metabolism to stabilize the T6SS MC in *A*. *baumannii*, encompassing a remnant of an ancestral but cryptic acyl synthase domain maintained in some related polyketide synthases.

**Fig 7:**
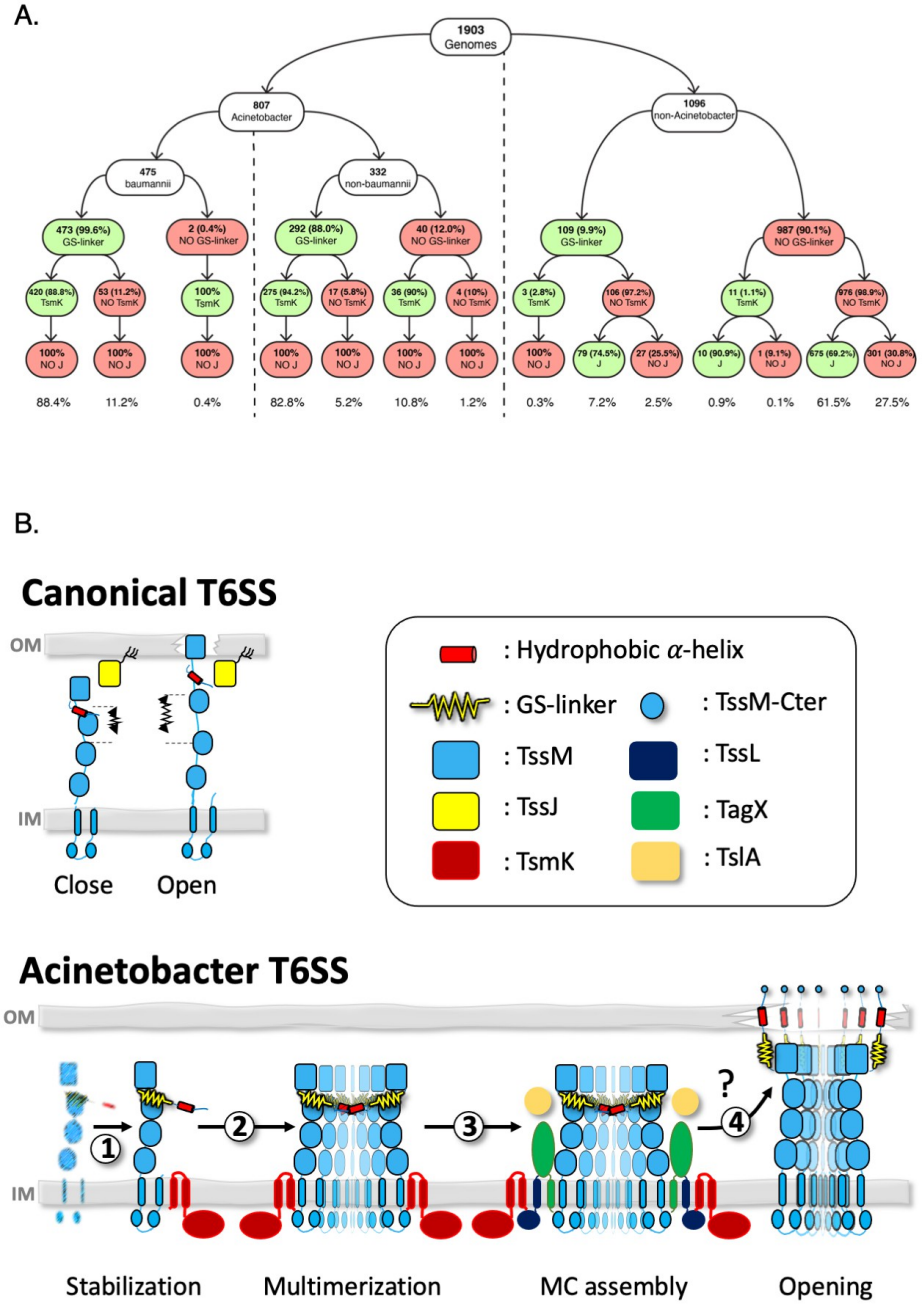
Model for the assembly of the *A*. *baumannii* T6SS MC. **(A)** Conditional probability tree of the co-occurrence of “GS-linker motif”, TsmK, and TssJ on genomes containing both a *tssB* and a *tssM* gene. Percentages inside the rounded rectangles represent the conditional probabilities after each branching. Percentages reported on the bottom are the joint probabilities of the different GS-linker -TsmK -TssJ configurations in *baumannii*, non-*baumannii* and non-*Acinetobacter* genomes. Rounded red and green rectangles represent the absence and the presence of one of the two genes (*tssJ* or *tsmK*) or the GS-linker motif, respectively. **(B)** *A*. *baumannii* T6SS offers a unique glimpse of molecular evolution showing how the absence of a major T6SS component (like TssJ, upper panel) could be compensated by small additional domain or co-opted protein (lower panel). **(1)** TsmK stabilizes the pillar of the membrane complex (MC), the protein TssM. **(2)** Helped by the GS-linker, TssM oligomerizes to assemble a mega-membrane complex with TsmK. **(3)** TssL, TagX and TlsA are recruited to form the *Acinetobacter* T6SS MC. **(4)** The molecular mechanism and dynamic of outer membrane anchoring and piercing by the MC is not yet known.

Our bioinformatic analysis evidence that TsmK, TssM GS-linker, and the absence of TssJ are three co-occurring events in the evolution of the *A*. *baumannii* T6SS. Two hypothesis can be drawn from this observation (Fig 7A). (1) This arrangement reflects a pure co-incidence and does not bring any information regarding the replacement of TssJ function. (2) Two elements, TsmK and TssM GS-linker, when combined substitute at least partially for the absence of TssJ. Indeed, we show that TsmK stabilizes the MC and the TssM GS-linker is crucial for the oligomerization of TssM periplasmic domain, two important actions that have been previously assigned to TssJ [19,29,39]. However, a yet-to-be identified function needs to be elucidated: the insertion of the MC into the outer membrane. We propose three possible scenarios: (1) the GS-linker could maintain the proper orientation of TssM β-strand domain for reaching and crossing the OM, as it has been observed for the EAEC TssM [19]. This is supported by the comparison between the AF2 models of TssM-CTD with ΔGS and the EAEC and WT structures (S6C Fig). (2) The GS-linker is a spring that allows the extreme C-terminal domain of TssM to reach out the OM. (3) Another protein fulfills the function of TssJ and will need further work to be identified. It could be a combination of these three possible situations.

Previous work has indirectly demonstrated that the C-terminal domain of TssM could be at least transiently exposed to the extracellular environment, where it could play a hypothetical function such as prey cell detection [19]. Remarkably, we observed here that fusing the bulky *sf*GFP to the C-terminal of *A*. *baumannii* TssM allowed us to monitor what could represent outward motions of this domain. Even though we did not directly prove the extracellular exposure of the C-terminally tagged *sf*GFP, the amplitude of its location when fused to the C-terminal of TssM strongly suggests that it could reach the extracellular milieu, as it has been proposed for Enteroaggregative *E*. *coli* TssM [19]. The visualization of the TssM C-terminal location in and out the outer membrane-label of *A*. *baumannii* probably reflects the close and open states of the T6SS OM channel, respectively. Together, these results point to the existence of a channel, large enough to allow the passage of the Hcp tube that is assembled by the T6SS apparatus and whose existence has been suggested [19,39] but whose experimental evidence remains scarce (Fig 7B). Our work on the *A*. *baumannii* MC represents a major advance to address the assembly of this postulated T6SS OM channel. However, we have not yet precisely identified the exact protein domain involved and future work will aim to determine its architecture and dynamic assembly.

Using live fluorescent microscopy, we observed that the *A*. *baumannii* MC forms dynamic foci that assemble and disassemble in a short period of time and coordinately with the sheath assembly process (see Fig 2C). This finding implies that upon contraction of the TssBC sheath and the firing of toxins into prey cells, the MC disassembles and then reassembles for a new round of elongation/contraction/injection events. Our observation is in contrast with other reported study showing that the Enteroaggregative *E*. *coli* or *Serratia marcescens* T6SS MCs assemble stable and static fluorescence foci that are re-used for several rounds of elongation/contraction event [19,40]. Nevertheless, works on *S*. *marcescens* MC have shown that an excess pool of TssL, as compared to cytosolic TssB-sheath, exists in the inner membrane together with TssJ distributed evenly in the outer membrane (OM) [40]. All these seemingly separated pools could congregate and assemble a functional T6SS apparatus at specific envelope sites. One can envisage that this regulated process (*i*) avoids the assembly of an energy-consuming nanomachine when it is not needed, (*ii*) drives the assembly of the T6SS where it is needed and (*iii*) prevents the OM leakage and/or the continuous inward diffusion of antibiotics through the T6SS channel. In line with our discovery, a study on *V*. *cholerae* T6SS has demonstrated that the recruitment of effectors is critical for T6SS assembly, representing an effective onboard checking mechanism that ensures effectors are loaded in place to prevent futile secretion [41]. Altogether, these results suggest that upon T6SS firing, the C-terminus of TssM translocates from the periplasm to the exterior of the cell and that ClpV activity is important to reversing this as in its absence when T6SS can fire, but not recycle, the average location shifts.

In most proteobacterial T6SS MC the assembly is stabilized by TssJ membrane-anchoring proteins. In *A*. *baumannii* T6SS the absence of a membrane-anchoring protein raises the question of how MC stabilization occurs. We posit that the absence of a TssJ-like component precludes the fine tuning of temporal and local assembly of the MC. Instead, we propose that in *A*. *baumannii*, *ad hoc* cycles of assembly/disassembly regulate the opening of the T6SS nanomachine to coordinate the OM-breaching mechanism tightly with the firing of the Hcp arrow. These steps, that ensure the building of an open T6SS channel are all levels of control that are part of a strategy used by *A*. *baumannii* to preserve the barrier function of the OM, particularly in an aggressive environment where antibiotics and other antimicrobial peptides are legion. This observation could have major impacts on the understanding of T6SS functioning and its adaptation to pathogen’s lifestyle. *A*. *baumannii* has been classified the number one “public enemy” in the ESKAPE group by the World Health Organization due to its multi-resistance to antibiotic chemotherapy. Although, we don’t know yet the direct or indirect implication of T6SS in *A*. *baumannii* virulence, certain strains repress their T6SS in parallel to acquire antibiotic-resistance mechanisms. Indeed, previous works demonstrated that *A*. *baumannii* blocks its T6SS from functioning when in presence of antibiotics owing to the utilization of negative transcriptional regulators encoded on mega plasmids [42]. Interestingly, the expression of virulence factors embedded in the bacterial envelope (T3SS and flagella) increases outer-membrane permeability and sensitivity to envelope stress in *Salmonella Typhimurium* [32]. The repression of these membrane nanomachines restores stress resistance. This observation raises the possibility that T6SS functioning could have deleterious effect on the development of antibiotic resistance strategy by the pathogen. A recent study has demonstrated that the functioning of the T6SS renders *A*. *baumannii* incapable of coping the phagocytic cell-mediated oxidative stress due to inadequate uptake of Mn^2+^ [43]. Interestingly, the authors identified a *bona fide* sRNA that mediates the post-transcriptional repression of T6SS by acting on the mRNA of *tssM*. Thus, the presence of the membrane complex is likely to impair the Mn^2+^ balance and in turn prevent the oxidative stress response of *A*. *baumannii*. One can ask if the mere presence or action of the T6SS renders *A*. *baumannii* more susceptible to antibiotic? This observation can be reconciled considering our finding: the MC could penetrate the OM and assemble a channel large enough to fire the T6SS arrow. In this scenario, the GS-linker could play a crucial role as the main driver of MC oligomerization (Fig 7). Interestingly, the proposed position of the GS-linker in *A*. *baumannii* TssM sequence places it up-stream of the hydropathic helix observed in the structure of the EAEC TssM [19]. Although the structure of the *A*. *baumannii* TssM remains to be experimentally determined, some speculations can be drawn on the possible function of the GS-linker. If the C-terminal TssM helix could act as a membrane penetration device, eg, by forming channels. The flexibility and the length of the GS-linker is an important property to deliver the helix to the membrane, as is observed in GLY mutagenesis and GS-linker deletion. In this context, the molecular understanding of the specificity of the *A*. *baumannii* T6SS should bring a wealth of information on its mechanism of action and notably on its integration into the bacterial cell envelope. One can envisage that the particular membrane crossing mechanism we propose here creates such destabilization of the lipid bilayer that it renders the cell susceptible to anti-bacterial agent. In line with this hypothesis, several studies indicate that T6SS is repressed in various ways by clinical isolates of *A*. *baumannii* [42,44]. In the future, our findings could inspire new biomimetic membrane destabilizers that prevent the expansion of the antibiotic-resistance arsenal of *A*. *baumannii*.

Our visualization of the motion of the TssM-CTD combined with previous studies showing that loops within the last β-strand domain of TssM are extracellularly exposed when T6SS fires toxins [19], altogether point towards a channel function for this component of the nanomachine. The TssM-CTD holds a conserved C-terminal peptide composed of hydrophobic residues with tendency to form a helical structure. This secondary structure has been visualized in the cryo-EM structure of the EAEC MC [39] and only predicted in *A*. *baumannii* TssM (Fig 7). The TssM CTD helix is reminiscent to similar structural features described in other transenvelope transport systems. The TraF/VirB10 homologue from the T4SS membrane core complex harbours an α-helical antenna [45], where VirB10 subunits project each a-helical bundle to form a highly unusual outer-membrane channel [46]. The protein Wza, a component of the capsular polysaccharide (CPS) biosynthesis pathway that export lipid-linked polysaccharides across the periplasm and OM to form an extracellular capsule, inserts a C-terminal *α*-helix into the OM to assemble an oligomeric channel [47]. In this latter example, the complex traverses the OM using an *α*-helical barrel, formed by eight C-terminal amphipathic helices, one from each monomer, that assemble into a cone-shape channel *via* oligomerization. Together our findings point to a structural parallel between TssM and VirB10/Wza, and thus provide support for the hypothesis that the C-terminal *α*-helices from each TssM monomer could oligomerize and assemble a genuine OM channel (Fig 7). In most T6SS bacteria the insertion of the TssM-CTD helix in the outer membrane could be piloted by the TssJ lipoprotein. This raises the question on how this insertion happens in the absence of a TssJ-homolog like in *A*. *baumannii*? Interestingly, we found a conserved Glycine-Serine rich peptide (GS-linker) right before the potential C-terminal amphipathic helix in *Acinetobacter*. This latter feature together with the absence of TssJ and the presence of TsmK are co-occurring events that have been co-selected in *Acinetobacter* genomes. Ala substitution of the first three Gly residues or deletion of the GS-linker show that they play a crucial role in TssM multimerization and the overall T6SS functioning, conceivably implicated in driving and stabilizing the helix-helix association for channel formation within the OM. Using an *in-silico* approach, we suggested that deletion of GS-linker could trigger a tectonic rearrangement of the beta-domain relative to the last alpha-domain (S6C Fig). GS-linkers are known to act as flexible molecular spacer ensuring efficient coupling between interacting protein domains or partners [48]. Collectively, we propose that the absence of TssJ in *Acinetobacter* is compensated by the presence of the GS-linker that somehow allows the CTD helix to reach the outer membrane for ensuring both TssM oligomerization and channel assembly. Future studies will need to determine the actual structure of *A*. *baumannii* TssM and the role of this GS-linker in the assembly of the T6SS channel. The study of TssM-CTD and the discovery of these motifs opens the path for deeper understanding of the T6SS membrane complex. Both the GS-linker and the CTD helix could be used as a fundamental tool as well as a new therapeutic target.

## Material and methods

### Bacterial strains and growth conditions

The strains, plasmids and nucleotides used in this study are listed in S1 Table. *E*. *coli* and *A*. *baumannii* ATCC 17978 strains were grown in LB medium at 37°C with shaking. The antibiotics kanamycin (40 μg/ml), streptomycin (50 or 100 μg/ml), chloramphenicol (30 μg/ml), rifampin (100 μg/ml), gentamicin (10 or 50 μg/ml), carbenicillin (400 μg/ml) and tetracycline (5 μg/ml) were added where necessary.

### Plasmid construction

PCRs were performed using Q5 high-fidelity DNA polymerase (New England Biolabs). Restriction enzymes from New England Biolabs was used according to the manufacturer’s instructions. Custom oligonucleotides were synthetized by IDT and are listed in S1 Table. *A*. *baumannii* 17978 chromosomal DNA were used as templates for PCRs. *E*. *coli* DH5α was used for cloning procedures. Briefly, the sequence encoding full-length gene was PCR-amplified using primers (FV and RV). The PCR introduced a 5’ and a 3’ restriction sites and usually a C-terminal TAG-extension. Restrictions were performed at 37°C with 1X NEBuffer r3.1 and the ligation at 16°C with T4 DNA Ligase Buffer (10X). Product was then transformed into competent *E*. *coli* DH5α, and recombinant strains were selected on the appropriate antibiotic. All the plasmids were engineered by restriction cloning except for the pVRL2-[TssM(GS^G/A^)^His^], pVRL2-[TssM(ΔGS)^His^], pACYC-[TssM_930-1228_(ΔGS)^Strep^] and pRSF-[^HIS^TssM_930-1228_(ΔGS)^FLAG^]. The pVRL2-[TssM(GS^G/A^)^His^], pVRL2-[TssM(ΔGS)^His^], pACYC-[TssM_930-1228_ (ΔGS)^Strep^] and pRSF-[^HIS^TssM_930-1228_(ΔGS)^FLAG^] vector plasmids were engineered by a two-fragments sequence- and ligation-independent cloning (SLIC) strategy [49,50]; and the pRSF- [TssM ẞ^HIS^Ab(ΔGS), pRSF-[TssJ^STREP^EAEC] and pRSF-[TssM ẞ^HIS^EAEC+TssJ^STREP^EAEC] were constructed by megapriming. Briefly, each DNA fragments were amplified by PCR using two pairs of oligonucleotides (FW1/RV1 and FW2/RV2). PCR products were digested (DpnI), cleaned (Macherey-Nagel PCR cleaning kit), and mixed with the T4 DNA polymerase and its buffer (NEBuffer r2.1) at room temperature. The reaction was stopped after 2 min 45 s, and the mixture was put on ice. The annealing product was transformed into competent *E*. *coli* DH5α, and recombinant strains were selected on the appropriate antibiotic. The pCter-peptide is a synthetic plasmid designed by Genecust that encompasses the DNA sequences encoding for an in frame *A*. *baumannii* DsbA signal sequence fused to TssM C-terminal from amino acid 1166.

### Construction *of A*. *baumannii* mutants

The primers used in this study are listed in S1 Table in the supplemental material. Mutants were constructed as described previously [51]. Briefly, an antibiotic resistance cassette was amplified using 2 sets of primers. The first one (A-primer) was composed of ~55-bp oligonucleotide homology to the flanking regions of the targeted gene with an additional 3’ 18 to 25 nucleotides of homology to the FRT site-flanked kanamycin resistance cassette from plasmid pKD4. The second (B-primer) was composed of ~55-bp oligonucleotide homology to the flanking regions of the targeted gene in 5’ of the previously amplified region with an additional 3’ 18 to 25 nucleotides of homology to the A primer. The PCR product was electroporated into a competent T6+ *A*. *baumannii* ATCC 17978 carrying pAT04, which expresses the RecAb recombinase. Mutants were selected on 12 ug/mL kanamycin, and integration of the resistance marker was confirmed by PCR. To remove the kanamycin resistance cassette, electrocompetent mutants were transformed with pAT03, which expresses the FLP recombinase, to remove the FRT-flanked resistance cassette. Additional mutations were made by repeating this process, and PCR, sequencing and complementation were used to confirm the validity of all the mutants.

### SDS-PAGE, protein transfer, immunostaining, and antibodies

SDS–PAGE was performed on Bio-Rad Mini-PROTEAN systems using standard protocols. For immunostaining, proteins were transferred onto 0.2-μm nitrocellulose membranes (Amersham Protran) Immunoblots were probed with primary antibodies and goat secondary antibodies coupled to alkaline phosphatase and developed in alkaline buffer in presence of 5-bromo-4-chloro-3- indolylphosphate and nitro-blue tetrazolium. The anti-HA (HA-7 clone, Sigma Aldrich), anti-Flag (Sigma Aldrich, A2220-1ml or F3165-.2MG), anti-StrepII (Bio-Rad), anti VSV-G (Sigma Aldrich, SAB4200695-100UL), anti-6His (Sigma Aldrich, SAB1305538-400UL), anti-StrepII (Biorad MCA2489), anti-GFP (Thermofischer, MA5-15256), RNA-pol II (Ozyme 664906) monoclonal antibodies, rabbit polyclonal mcherry antibodies (OriGene TA150125) mouse secondary antibodies (Interchim 115-055-003) and rat secondary antibodies (Interchim115-055-045) were purchased as indicated. Synthetics polyclonalrabbit antibodies were designed by GeneScript for Hcp (epitope: SVGGHTAERVEHSDC) and TssM detection (epitope: CAPAQAAAPAKTENP).

### Hcp secretion

Overnight cultures were back diluted in fresh LB medium to an OD600 of 0.05 and grown at 37°C with shaking (induction of the pVRL2 plasmid expression was performed by adding 0.1% of arabinose 1h after starting or by adding 1% arabinose and induction of the pVRL1 plasmid expression was performed by adding 2mM of ITPG 1h after starting, since the beginning of the culture when necessary) until they reached an OD600 of 1.5 approximately. The cells were then pelleted by centrifugation and resuspended in Laemmli buffer with β-mercaptoethanol to a final OD600 of 0.02 while the supernatant fraction was centrifuged twice again (as above) to pellet residual cells. Supernatant proteins were subsequently precipitated with trichloroacetic acid 10% and resuspended in Laemmli buffer with β-mercaptoethanol to a final OD600 of 0.1. Detection of Hcp in normalized samples of whole cells and culture supernatants by Western blotting was performed as described previously, with an anti-Hcp, anti-RNA polymerase antibodies, which were used as a lysis control or an anti-TssM, used to control the expression level of the protein in mutants complemented with different plasmids.

### Bacterial killing

Cultures of *A*. *baumannii* and *E*. *coli MG1655R* were grown overnight, and the *E*. *coli* cells were washed three times with fresh LB to remove rifampin. The cells were then resuspended to an OD600 of 1.0, 100 μl of *E*. *coli* was mixed with 10 μl of *A*. *baumannii*, and a 10 μl sample was spotted onto a dry LB agar plate. After a 4 h incubation at 37°C, spots were cut from the agar and resuspended by mixing with 500 μl of LB broth. This mixture was serially diluted 10-fold, and dilutions were plated onto rifampin-containing LB agar to determine the number of *E*. *coli* CFU remaining. Controls consisted of *E*. *coli MG1655R* mixed with rifampin sensitive LB medium. Experiments were performed twice in technical triplicate. Competitions for these experiments were done at a (1:10) *A*. *baumannii* to *E*. *coli* ratio, and *E*. *coli* bacteria were enumerated on agar plates containing rifampicin.

### Purification of soluble protein for interaction studies

Plasmids expressing the genes combination of interest were co-transformed into *E*. *coli* BL21(DE3). Cells were grown at 37°C in lysogeny broth (LB) to an OD600 ~ 0.8 and the expression of the TssM_930-1228_ genes was induced with 1.0 mM IPTG for 3 hours at 37°C. Cell pellets were resuspended in 50 mM Tris-HCl pH8.0, 150 mM NaCl, 1 mM EDTA and supplemented with 100 μg/ml of DNase I, 100 μg/mL of lysozyme and 10 mM of MgCl2 (lysis buffer). The soluble fraction was then collected by centrifugation at 41 000 g for 45 min. The supernatant was loaded onto a 1-ml Strep-Trap-HP (GE Healthcare) column and then washed with 50 mM Tris-HCl pH 8.0, 150 mM NaCl (soluble affinity buffer) at 4°C. The co-purified proteins were eluted in the soluble affinity buffer supplemented with 2.5 mM desthiobiotin (Sigma Aldrich). The lysate, flow through, wash and elution fractions were collected, resuspended in Laemmli loading buffer supplemented with 300 mM 2-Mercaptoethanol, heated for 10 min at 96°C prior to analyses by SDS-PAGE and immunoblotting.

### Purification of full-length membrane protein for interaction studies

Plasmids expressing the genes combination of interest were co-transformed into *E*. *coli* BL21(DE3) and cells were treated as described below. Cells were grown at 37°C in lysogeny broth (LB) to an OD600 ~ 0.8 and the expression of the genes was induced with 1.0 mM IPTG for 3 hours at 37°C. Cell pellets were resuspended in 50 mM Tris-HCl pH8.0, 50 mM NaCl, 1 mM EDTA and supplemented with 100 μg/mL of DNase I, 100 μg/mL of lysozyme, EDTA-free protease inhibitor (Roche) and 10 mM of MgCl2 (lysis buffer). Cells were broken using an Emulsiflex-C5 (Avestin) and clarified by ultracentrifugation at 10 000 g for 15 min. The membrane fraction was then collected by centrifugation at 41 000 g for 45 min. Membranes were mechanically homogenized and solubilized in 50 mM Tris-HCl pH 8.0, 50 mM NaCl, 0.5% w/v n-dodecyl-β-D-maltopyranoside (DDM, Anatrace), 0.75% w/v Decyl Maltose Neopentyl Glycol (DM-NPG, Anatrace), at 4°C overnight. The suspension was clarified by centrifugation at 35 000 g for 35 min. For His-tag affinity, the supernatant was loaded onto a 5 mL His-Trap-HP (GE Healthcare) column equilibrated in affinity buffer (50 mM Tris-HCl pH 8.0, 50 mM NaCl, 0.05% w/v DM-NPG) supplemented with 20 mM imidazole. The column was then washed using the affinity buffer supplemented with 50 mM imidazole and protein complexes were eluted in the same buffer supplemented with 200–250 mM imidazole. For strep-tag affinity, the supernatant was loaded onto a 5 mL Strep-Trap-HP column (GE Healthcare), washed with affinity buffer, and eluted in affinity buffer supplemented with 2.5 mM desthiobiotin (Sigma Aldrich). The lysate, flow through, wash and elution fractions were collected, resuspended in Laemmli loading buffer supplemented with 300 mM β-mercaptoethanol, heated for 10 min at 96°C prior to analyses by SDS-PAGE and immunoblotting.

### Native purification of *A*. *baumannii* TssM

*A*. *baumannii*::*tssM*^STREP^ strain was transformed with the pVRL2-*tsmK*^HA^ plasmid. Growth was performed in LB medium at 37°C and induction was performed with 2mM IPTG 1h after starting, during 5H. See above for a full description of the procedure (membrane extraction and purification on STREP column).

### Inner and outer membrane separation using sucrose gradient

The cells were pelleted, 250 OD_600_ were resuspended in the lysis buffer, and then broken using an Emulsiflex-C5 (Avestin) and clarified by ultracentrifugation at 10 000 g for 15 min. The crude membrane fraction was isolated by ultracentrifugation at 100 000 g for 45 min and then resuspend in 0.5 mL. Outer and inner membranes were separated by differential centrifugation using a continuous sucrose gradient by using a peristatic pump (the gradient was produced by mixing two solutions, one with 35% and the other with 60% sucrose). We ultracentrifuge the tubes in a swing-out rotor ON at 274 000 g at 4°C. After centrifugation, 750ul samples were taken from the top to the bottom of the gradient.

### Homologous search

The search of TsmK homologs was performed by BLASTp [53] based on the Uniref90 database [52] using as a query the TsmK sequence of *A*. *baumannii* ATCC 17978 strain. To infer the presence of a particular gene on a specific genome, we look for homologs of this gene on the given genome using BLASTp. When inferring TsmK presence, we used the already mentioned *A*. *baumannii* ATCC 17978 TsmK sequence as a query. In the case of TssM, Tssj, and TssB, we queried the TssM, TssJ, and TssB sequences from a selected list of pathogenic T6SSs (S2 Table). Homologous sequences whose blast e-value was higher than 1-e20 or whose query coverage was below 60% were discarded and hence not used to determine the presence of a particular gene on a given genome. Uniref90 and the different genomes were converted to blast databases using the makeblastdb application [53].

### TaxID and genome assignment

To assign a TaxID to a given sequence, we used UniProt (UniProt Consortium 2021) annotations and the NCBI taxonomy database (UniProt Consortium 2021). Sequences that could not be assigned a TaxID were discarded. A given TaxID might have multiple subtree TaxIDs. When this happens, a unique genome could not be assigned to it. In this case, we discarded the TaxID and any sequence associated with it. EDirect NCBI tools [54] were used to download the unique genome associated with a TaxID with no subtree with multiple TaxIDs. *Acinetobacter* genomes were obtained from a list of 6041 *Acinetobacter*

TaxIDs collected from the NCBI taxonomy database. Apart from TaxIDs with multiple TaxIDs, the ones whose associated genome has less than 500 genes were discarded. As a result, we ended up with a set of 893 *Acinetobacter* genomes carrying a TssB gene (used as an indicator of a complete T6SS operon). To guarantee the diversity of the set of non-*Acinetobacter* genomes containing a *tssM* gene, we used the TssM sequences from the list of pathogenic T6SSs (S2 Table) as queries during a BLASTp search on Uniref90. The resulting homologs whose length was less than 50% or more than 150% of the query length were discarded. Next, we merged the remaining sequences and clustered them by 0.9 sequence identity using cd-hit [55]. Then, we assigned a unique TaxID and genome to each TssM homolog. Finally, we ended up with a set of 1096 non-*Acinetobacter* genomes carrying both a TssM and a TssB gene.

### Protein conservation

We used a previously developed closeness metric [31] to compute the conservation level of proteins of interest. As input, we used the TssM, TsmK, TssB, and TssK sequences obtained from the 893 *Acinetobacter* genomes aligned with Mafft [56]. In the case of only *A*. *baumannii*, we filter the sequences using the Uniprot annotations and the NCBI taxonomy database. *A*. *baumannii* TsmK models. Two transmembrane helices in the N-terminal region of *A*. *baumannii* TsmK (from I5 to W24 and from F36 to I57) were predicted using the TMHMM server [57]. The sequence of the transmembrane helices was not included in the structural modeling of *A*. *baumannii* TsmK (from Y58 to S470) performed by AlphaFold2 [58,59], TrRosetta [60] and RaptorX [26]. To identify consistent regions between the three TsmK models, we conducted a structural alignment using TMalign [61]. The structural homology search for the consistent model of TsmK was performed by the Dali server [27].

### Bioinformatic data analysis, visualization, and storage

To generate the multiple sequence alignment (MSA) figures (Figs 6B, S4H, S6A and S6B) and compute the residue conservation, we used JalView [62]. The molecular visualizations of TsmK models (Figs 4A, 4C, S4E and S4F) were rendered with UCSF Chimera [63]. MSA residue conservation was represented on a sequence logo plot using the WebLogo server (Fig 6B) [64]. Secondary structure topological diagrams (Fig 4B and 4D) were assembled with TopDraw [65]. All Python and shell scripts developed explicitly for this work are available with the initial genomic data (TsmK, TssM, TssJ, and TssB sequences) and the resulting homologous sequences at https://github.com/IFilella/AbAsaBTssM.

### TssM C-terminal “GS-linker motif” identification

We used four different amino acid segments to infer the presence of the GxxGxxxG motif in the TssM C-terminal domain: GxxxGxxxG, GxxGxxxG, GxxxGxxG, and GxxGxxG, where x stands for any of the 20 bacterial amino acids. To validate the presence of the GxxGxxxG motif, the last 100 residues of TssM should possess at least one of these segments. Importantly, these segments do not exclude longer segments with an additional intermediate region (xx or xxx) followed by a fourth Gly.

### Fluorescence microscopy

*Sample preparation*: Cells were spotted on thin pad supplemented with 3% agarose in PBS 1x, made inside a gene frame (Thermo Scientific- Gene Frame) taped to a microscope slide. An SR cover glasses (Marienfeld Superior—Precision cover glasses No. 1.5H) was then used to seal the preparation. Imaging was performed on a DeltaVision OMX SR Imaging system (GEHealthcare) with an Olympus PlanApo N 60× 1.42 NA oil immersion objective and liquid-cooled sCMOs cameras (IMM Microscopy Platform).

*Data analysis—Clusters density quantification and clusters per cell quantification*: Images were acquired using excitation at 488 nm during 500 ms at 100%T for sf-GFP and excitation at 568nm during 500 ms at 100%T for mCherry. For each acquisition a BrightField image was generated. The masks of cells were obtained directly from a brightfield image by thresholding Images were filtered with FFT band pass (1 to 5 pixels) with FIJI [66]. The fluorescent clusters were detected by local maxima algorithm in MicrobeJ plugin ([67]; http://www.microbej.com/) that yields the localization density maps and the cell histograms. The statistics of the number of clusters per cell were calculated with R (R Core Team (2023). _R: A Language and Environment for Statistical Computing_. R Foundation for Statistical Computing, Vienna, Austria. https://www.R-project.org/).

*Data analysis—TIRF*: Total Internal Reflection Fluorescence (TIRF) Imaging was realized using the RING-TIRF mode from DeltaVision OMX, with illumination angle around 80° and an Olympus PlanApo N 60× 1.49 NA oil immersion objective. Time-lapse recordings were analyzed with the following workflow: 1—projection of the mean of fluorescence channel. 2—detection of the cell masks with cellpose [68] with the pre-trained model “cyto2” 3—filtering of the image by a bandpass FFT filter of FIJI/ImageJ, settings = minimum = 1 pixel, max = 5 pixels 4—cell tracking and contour intensity analysis and creation of kymographs with the MicrobeJ plugin on the FFT filtered fluorescence channel. (Fig 5B) The images were then analyzed using ImageJ (http://imagej.nih.gov/ij/) and the MicrobeJ plugin (http://www.microbej.com/).

*Data analysis–Kymographs*: Dynamics of fluorescent clusters of the TssM protein. Time-lapse was generated by acquiring 61 images every 10 seconds using excitation at 488 nm during 100 ms at 15%T. The masks of cells were obtained from the projection of the mean fluorescence channel, with Cellpose [69] with the pre-trained model “cyto2”. In parallel fluorescence channel images were filtered with FFT band pass (1 to 5 pixels) with FIJI. Cell tracking, contour intensity analysis and creation of kymographs were then done with the MicrobeJ plugin on theses filtered images.

*Data analysis– 3DSIM*: Images were acquired using an excitation wavelength of 488 nm during 200 ms at 10% Transmission for the sfGFP reporter and an excitation of 568nm during 200 ms at 5%Transmission for the mCherry reporter. For each acquisition, images of 512 × 512 pixels (pixel size of 80 nm) were acquired with a sample thickness of 1 μm (9 sections, Δz = 125 nm) centered at the middle of the bacteria. At each z position, SI images (five phases at three angles) were acquired for 3D-SIM imaging. Image analysis was performed using Fiji (version 1.53c) to extract intensity profiles which were then processed with python (version 3.9.5). The best z position of each stack was first selected so that sfGFP-spot and the membrane appear clearly in the image. Then intensity profiles were wisely plotted through sfGFP-spot with an orientation perpendicular to the membrane and directed towards the interior of the bacteria. The size of each profile has been set at 20 pixels (800 nm). Finally, fifty profiles were recovered for each mutant. The distance between the TssM and Omp28 was then measured with python (version 3.9.5) by fitting a gaussian curve to each profile and calculating the difference between the green maximum peak and the red maximum peak. For the heat-map, the images were acquired using excitation at 488 nm during 500 ms at 100% Transmission. The cell segmentation of the bright-field images was done using machine learning [70]. The images were then analyzed using ImageJ (http://imagej.nih.gov/ij/) and the MicrobeJ plugin (http://www.microbej.com/). Box plot Fig 5E representing the distances measured between the TssM^sfGFP^ foci and the mCherry label in a WT, a *clpV* mutant and a *tssB-tssC* mutant. The difference in position is equivalent to the distance between the two observed fluorophores and was measured in n = 60 bacteria (WT_ *ΔclpV*: t 3.1331, df = 105.39, p-value = 0.00224; WT_ *ΔtssBΔtssC*: t = 0.024481, df = 100.92, p-value = 0.9805; *ΔclpV*_Δ*tssBΔtssC*: t = -2.6278, df = 95.461, p-value = 0.01002).

### Mass Spectrometry–Based Proteomics

The list of identified proteins provided in Fig 3E (right panel) was established from mass spectrometry–based proteomics on excised SDS-PAGE gel bands (duplicates) containing the purified proteins. In-gel trypsin digestion and MSMS analysis were performed on a Q-Exactive plus mass spectrometer (ThermoScientific) as previously described [71], but slight modifications were brought: tryptic peptides were separated by a Vanquish_Neo liquid nanochromatography (ThermoScientific) on a C18 column (Easy-Spray PepMap Neo, 1500bar 75 μm x 500 mm, 2 μm, 100 Ǻ, ThermoScientific) at 300nl/min by a linear gradient from 4% to 25% of mobile phase B (0.1% formic acid (FA) (vol/vol) / 80% acetonitrile (vol/vol)) in mobile phase A (0.1% FA (vol/vol)) for 90 min, then to 50% of B in 20 min followed by a chase at 99% B. Spectra were processed by the Proteome Discoverer software 3.0 SP1 (version 3.0.1.27 from ThermoFisher) using the Sequest HT algorithm and the Chimerys identification node with the database extracted from NCBI, *Acinetobacter baumannii* strain ATCC 17978 (Taxon ID 400667; 5376 entries, last modified 02/13/2023). In this study, proteins were identified at “high level” protein FDR confidence.

### Outer membrane integrity assays

The permeability of the outer membrane was analysed by using the sensitivity to the antibiotic vancomycin. Briefly, *E*. *coli* BL21was transformed by a series of pRSF-Duet plasmid encoding…. Next, a colony of *E*. *coli* BL21 bearing the different pRSF-Duet plasmids was inoculated into 5 mL of LB media and incubated overnight at 37°C with shaking at 200 rpm. The overnight culture was diluted with sterile M9 medium to an OD600 = 0.1 and incubated in a 96-well NunclonΔ surface plate without shaking at 37°C until the culture reached an OD600 = 0.5. The cells were harvested by centrifugation (15,000 x g for 2 min), washed twice with assay buffer (5 mM HEPES, 5 mM glucose, pH 7.2) and resuspended in assay buffer to a final OD600 = 1.

## Supporting information

S1 Movie *A*. *baumannii tssM-sfGFP* clusters are shows very dynamic. Movie illustrating the dynamics of clusters in tirf. tssM-sfGFP clusters appear and disappear rapidly and some cells where clusters are not well visible. On the left the fluorescent signal and on the right the FFT band pass filtered image 5–1 pixels (Fiji/imageJ processing). Time = 10 sec / frame accelerated 200X.
(MP4)

S1 Fig Monitoring T6SS MC function of accessory proteins. **(A)**Bacterial competition experiments. Table associated with the Fig 1C. survival of *E*. *coli* rifampicin-resistant after incubation with ATCC 17978 WT and several mutants. **(B)** Phenotypic complementation of *tsmK* mutation measuring the Hcp secretion. Dot blot probing for Hcp secretion in supernatants, by *A*. *baumannii* ATCC 17978 WT, Δ*tssM* and the mutant Δ*tsmK*, transformed with the plasmid control empty (pVRL1) or the plasmid overexpressing TsmK. The TsmK protein production was induced by 1 mM IPTG.
(TIF)

S2 Fig Monitoring T6SS MC assembly and the function of accessory proteins. **(A)**Western blot assays probing for Hcp secretion and RNA polymerase (RNAP) in whole-cell pellets (P) and supernatants (S), by *A*. *baumannii* ATCC 17978 wildtype (WT, parental strain) and several mutants. The RNAP was used as control. The supernatants of each strain of *A*. *baumannii* are isolated, concentrated and then analyzed by denatured 12.5%-polyacrylamide gel electrophoresis (PAGE). Immunodetected proteins are indicated on the right. Molecular weight markers (in kDa) are indicated in the left. **(B)** Fluorescence microscopy recordings showing different constructions aimed at fusing the fluorophore (sfGFP) to a protein of the membrane complex. The images show: the phase contrast (top) and the fluorescence (bottom). The positions of the foci are indicated by arrows. The scale bars are 1 μm. **(C)** Comparison of fluorescent cluster distribution of TssM in different mutant. Diagram comparing the number of foci in TssM-*sf*GFP and TssM-*m*Cherry. Left panel, histogram representation of cluster distribution of TssM-*m*Cherry. Right panel, histogram representation of cluster distribution of the main axis of the cell with a preferential accumulation at the poles of the cell in two different mutants. The different mutants do not show very marked differences. The polar accumulation is less important in the d-ClpV mutant (n > 1000 cells for each group from two biological replicates). **(D)** Row data of the number of fluorescent TssM^sfGFP^ foci in different mutants **(E)** Non-polar effect of mutation on TssM expression. Western blot assays probing for the TssM^H^ and the EF-Tu expression in whole cell by *A*. *baumannii* ATCC 17978. The EF-Tu was used as control. The samples were analyzed by denatured 12.5%-acrylamide polyacrylamide gel electrophoresis (PAGE) and immunodetected with a Alexa Fluor Anti-Fluorescein (FITC) anti ET-Tu (FITC) and a fluorescent antibodies, λ_exitation_ = 488 nm. Immunodetected proteins are indicated on the right. Molecular weight markers (in kDa) are indicated in the left. **(F)** Time-lapse fluorescence microscopy recordings showing localization and dynamics of the ^*m*Cherry^ClpV and TssM^*sf*GFP^ fusions proteins (see Fig 2 for full legend). Two independent colocalization events are shown.
(TIF)

S3 Fig (A) Topology of TsmK using TMpred (https://topcons.cbr.su.se) (upper panel) or using Protter (https://wlab.ethz.ch/protter/#) (lower panel). (TIF)

S4 Fig Predicted structure and conservation of TsmK. **(A)** Conservation level comparison between TssM, TssB, TssK, and TsmK in *Acinetobacter* and in *A*. *baumannii*. **(B)** Sequence analysis of 116 TsmK homologs. Distinct proteobacterial genuses are reported as colored circles. The red vertical and horizontal dashed lines on the left graph represent the e-value (1e-20) and coverage thresholds (0.6), respectively, and are used to determine if a given homolog is a TsmK sequence (upper left quadrant). **(C)** A conditional tree describing the co-occurrence of TssM and TsmK on the genomes where the TsmK and TsmK-similar sequences were found. Zero and one, respectively, indicate the absence and the presence of TssM or TsmK. **(D)** Co-occurrence of TssM and TsmK in *Acinetobacter* genomes with a complete T6SS operon. **(E)** Structural superimposition of the three structural models of *A*. *baumannii* TsmK. **(F)** TsmK model and the structure of the Ketoacyl synthase. Regions that are inconsistent between AlphaFold2, trRosetta, and RaptorX are represented with transparent colors. **(G)** Structure of the Ketoacyl synthase domain from Acyltransferase type I polyketide synthase (PKS) (PDB 4TKT). **(H)** Structure-based sequence alignment of the Ketoacyl synthase structure 4TKT and the TsmK model. The conserved catalytic residues of the Ketoacyl synthase are highlighted with black squares.
(TIF)

S5 Fig Subcellular position of the TssM-CTD. **(A)** Fluorescent clusters of the TssM protein observed in TIRF. Image associated with the Fig 5A. Projection of the average in a part of a full field of microscope acquisition. The corresponding cells have been circled in blue. Scale: 2 μm. **(B-C)** Schematic representation of the different stages leading to the identification of the subcellular position of the TssM foci. For each channel (sfGFP and mCherry) a line is drawn from which is created a Gaussian representing the intensity of fluorescence, depending on the position (right panel). The positions of the green foci (TssM) and the outer membrane (Omp28) are thus determined by the x axis of the maximum value of their respective Gaussian. The difference between the position of the green foci (x) and the red membrane (y) is calculated in order to determine the distance between the two marked regions (in absolute value) as well as the subcellular location of the C-terminal of TssM (negative results: the foci is “outside” the cell; positive results: the foci is “inside” the cell) (left panel). On the right panel, structured illumination microscopy (SIM) images of the C-terminal TssM-sfGFP regarding the Omp28-mCherry label. Scale = 0.1 μm. On the left panel, an example of an intensity profile (blue) is shown. Example of Gaussian representing the intensity sfGFP and mCherry fluorescence related to one green foci.
(TIF)

S6 Fig Sequence and structural analysis of *A*. *baumannii* TssM. **(A)** MSA of *A*. *baumannii* and other proteobacterial species TssM C-terminal domain (from T987 to P1274 of *A*. *baumannii* TssM). Conserved regions were highlighted with a red square. The region of interaction between EAEC TssM and TssJ (PDB 4Y7O) was highlighted with a purple square. Residue conservation of the GxxGxxxGxxG motif found in the C-terminal domain of TssM for *Acinetobacter* was highlighted with grey dotted lines. **(B)** Multiple sequence alignment of *A*. *baumannii* TssM with the EAEC TssM (PDB 6hs7) to generate the structural model of *A*. *baumannii* TssM periplasmic domain. **(C)** Structural models for the TssM-CTD. (a) the structure of EAEC sequence. The region corresponding to the GS-linker of the Ab sequences is highlighted in green and boxed. (b,c) the structure of the *A*. *baumannii* sequence computed with AlphaFold (AF) 2.3.1 (cyan), and the *A*. *baumannii*-sequence with GS-linker deletion (blue) is aligned on the EAEC structure (gray), the GS-linker is colored in green and boxed. (d,e) 5 AF models for the Ab sequence colored using pLDDT score (red>90, blue<50). **(D)** Lower panel, western blot assays probing for TssM (WT and mutatants) production in whole cell lysates. The samples were subjected to denaturing 10%-polyacrylamide gel electrophoresis (PAGE) and immunodetected with synthetics polyclonal *A*. *baumannii-*TssM antibody.
(TIF)

S1 Table Strains, plasmids and oligonucleotides used in this study. (PDF)

S2 Table TssM sequences from pathogenic T6SS. (PDF)
