# Supplementary figures and images for: Acinetobacter type VI secretion system comprises a non-canonical membrane complex
Source: PLoS Pathog. 2023 Sep 28;19(9):e1011687. doi: 10.1371/journal.ppat.1011687 (PMC10564176; doi:10.1371/journal.ppat.1011687)

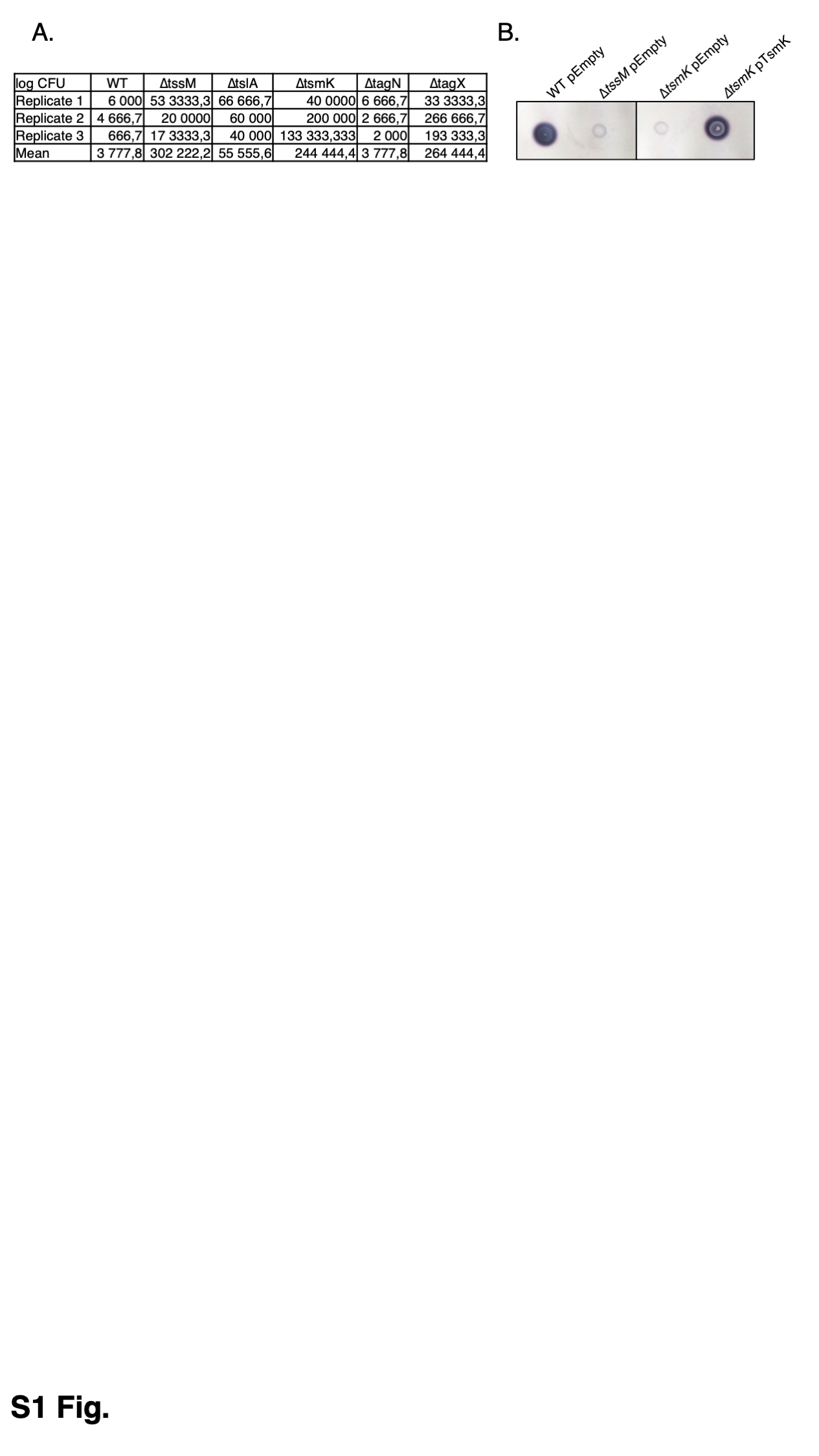

Supplement: S1 Fig — (A)Bacterial competition experiments. Table associated with the Fig 1C. survival of E. coli rifampicin-resistant after incubation with ATCC 17978 WT and several mutants. (B) Phenotypic complementation of tsmK mutation measuring the Hcp secretion. Dot blot probing for Hcp secretion in supernatants, by A. baumannii ATCC 17978 WT, ΔtssM and the mutant ΔtsmK, transformed with the plasmid control empty (pVRL1) or the plasmid overexpressing TsmK. The TsmK protein production was induced by 1 mM IPTG. (TIF) [file ppat.1011687.s002.tif]

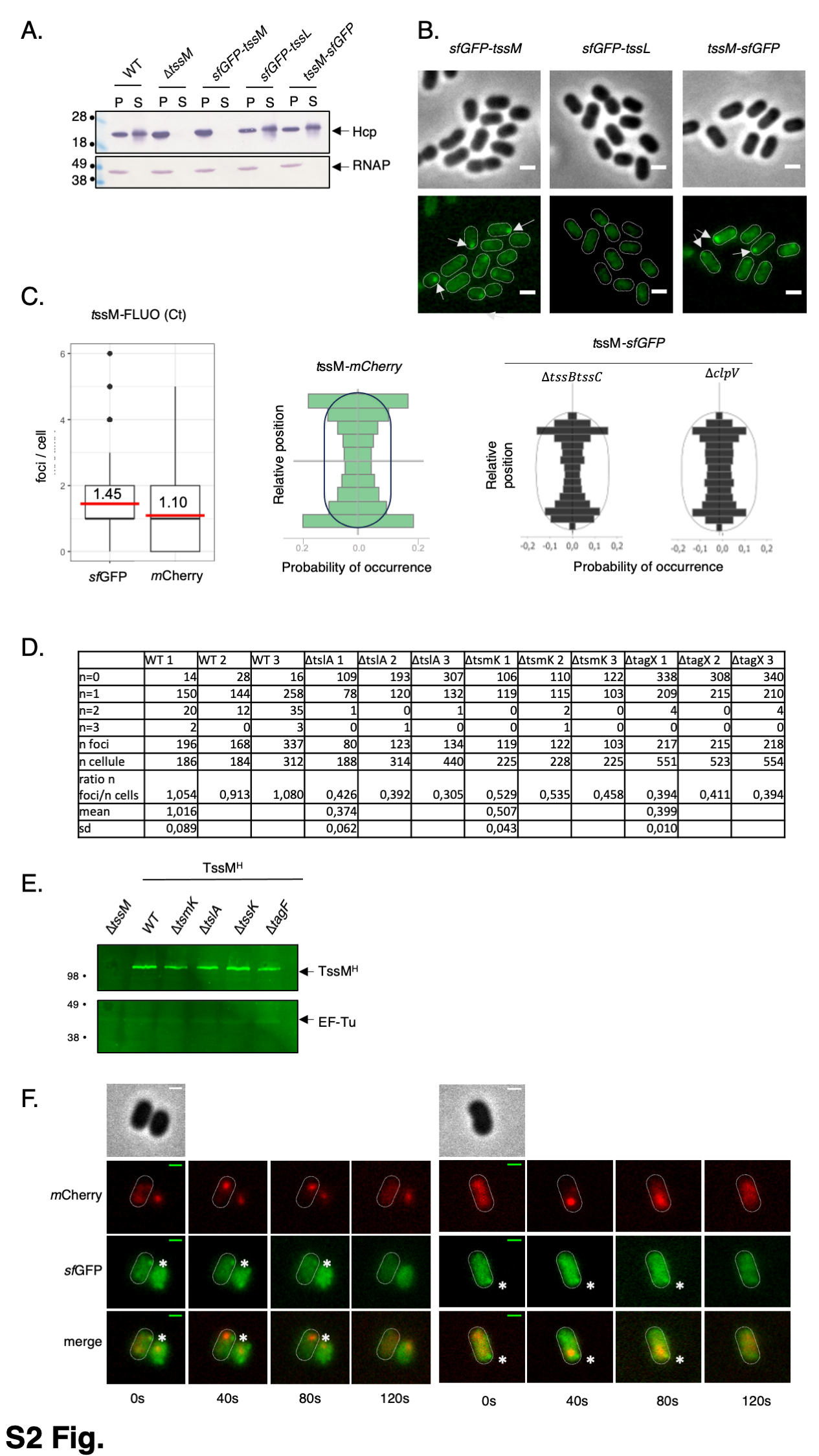

Supplement: S2 Fig — (A)Western blot assays probing for Hcp secretion and RNA polymerase (RNAP) in whole-cell pellets (P) and supernatants (S), by A. baumannii ATCC 17978 wildtype (WT, parental strain) and several mutants. The RNAP was used as control. The supernatants of each strain of A. baumannii are isolated, concentrated and then analyzed by denatured 12.5%-polyacrylamide gel electrophoresis (PAGE). Immunodetected proteins are indicated on the right. Molecular weight markers (in kDa) are indicated in the left. (B) Fluorescence microscopy recordings showing different constructions aimed at fusing the fluorophore (sfGFP) to a protein of the membrane complex. The images show: the phase contrast (top) and the fluorescence (bottom). The positions of the foci are indicated by arrows. The scale bars are 1 μm. (C) Comparison of fluorescent cluster distribution of TssM in different mutant. Diagram comparing the number of foci in TssM-sfGFP and TssM-mCherry. Left panel, histogram representation of cluster distribution of TssM-mCherry. Right panel, histogram representation of cluster distribution of the main axis of the cell with a preferential accumulation at the poles of the cell in two different mutants. The different mutants do not show very marked differences. The polar accumulation is less important in the d-ClpV mutant (n > 1000 cells for each group from two biological replicates). (D) Row data of the number of fluorescent TssMsfGFP foci in different mutants (E) Non-polar effect of mutation on TssM expression. Western blot assays probing for the TssMH and the EF-Tu expression in whole cell by A. baumannii ATCC 17978. The EF-Tu was used as control. The samples were analyzed by denatured 12.5%-acrylamide polyacrylamide gel electrophoresis (PAGE) and immunodetected with a Alexa Fluor Anti-Fluorescein (FITC) anti ET-Tu (FITC) and a fluorescent antibodies, λexitation = 488 nm. Immunodetected proteins are indicated on the right. Molecular weight markers (in kDa) are indicated in the left. (F [file ppat.1011687.s003.tif]

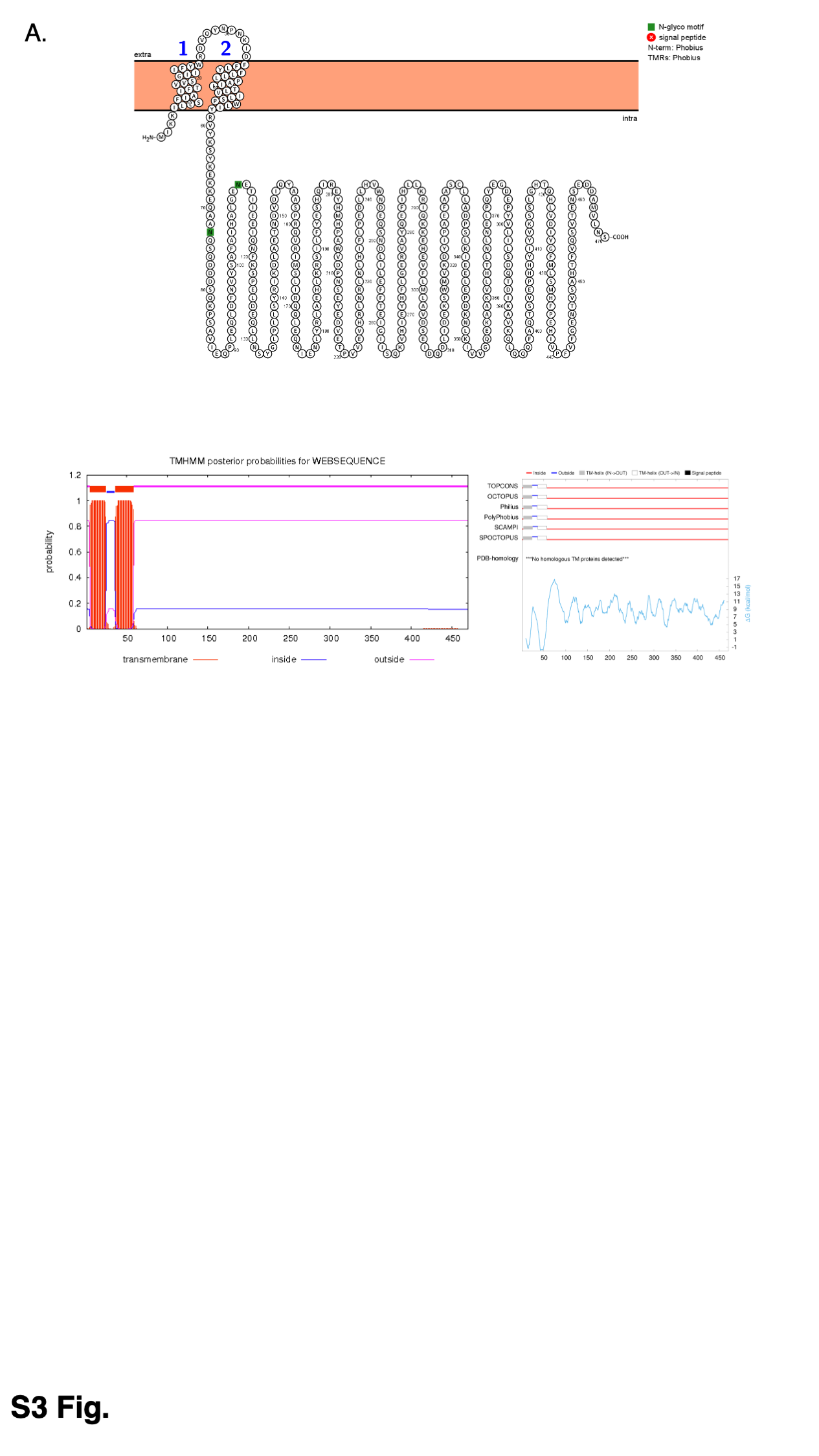

Supplement: S3 Fig — (TIF) [file ppat.1011687.s004.tif]

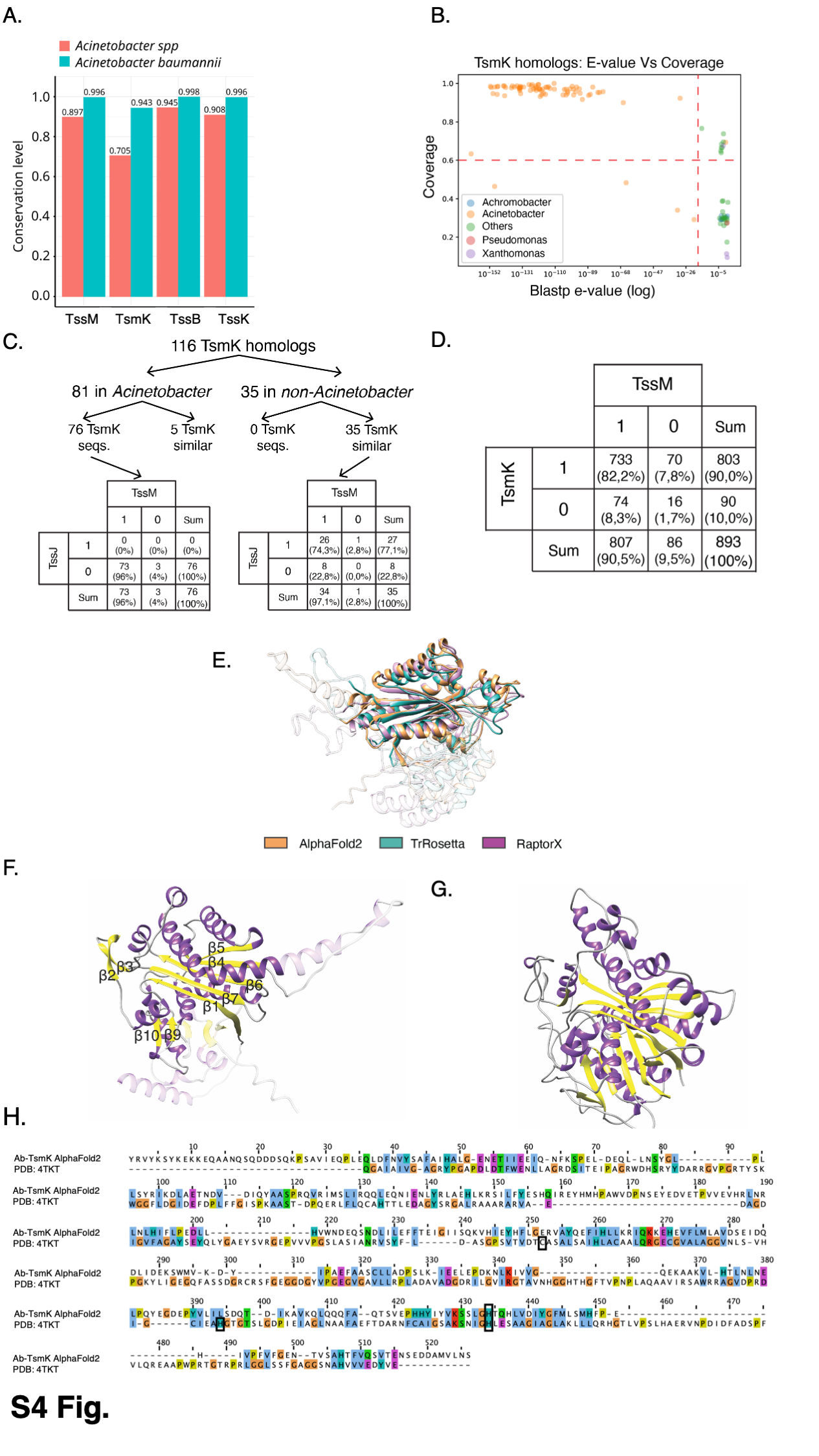

Supplement: S4 Fig — (A) Conservation level comparison between TssM, TssB, TssK, and TsmK in Acinetobacter and in A. baumannii. (B) Sequence analysis of 116 TsmK homologs. Distinct proteobacterial genuses are reported as colored circles. The red vertical and horizontal dashed lines on the left graph represent the e-value (1e-20) and coverage thresholds (0.6), respectively, and are used to determine if a given homolog is a TsmK sequence (upper left quadrant). (C) A conditional tree describing the co-occurrence of TssM and TsmK on the genomes where the TsmK and TsmK-similar sequences were found. Zero and one, respectively, indicate the absence and the presence of TssM or TsmK. (D) Co-occurrence of TssM and TsmK in Acinetobacter genomes with a complete T6SS operon. (E) Structural superimposition of the three structural models of A. baumannii TsmK. (F) TsmK model and the structure of the Ketoacyl synthase. Regions that are inconsistent between AlphaFold2, trRosetta, and RaptorX are represented with transparent colors. (G) Structure of the Ketoacyl synthase domain from Acyltransferase type I polyketide synthase (PKS) (PDB 4TKT). (H) Structure-based sequence alignment of the Ketoacyl synthase structure 4TKT and the TsmK model. The conserved catalytic residues of the Ketoacyl synthase are highlighted with black squares. (TIF) [file ppat.1011687.s005.tif]

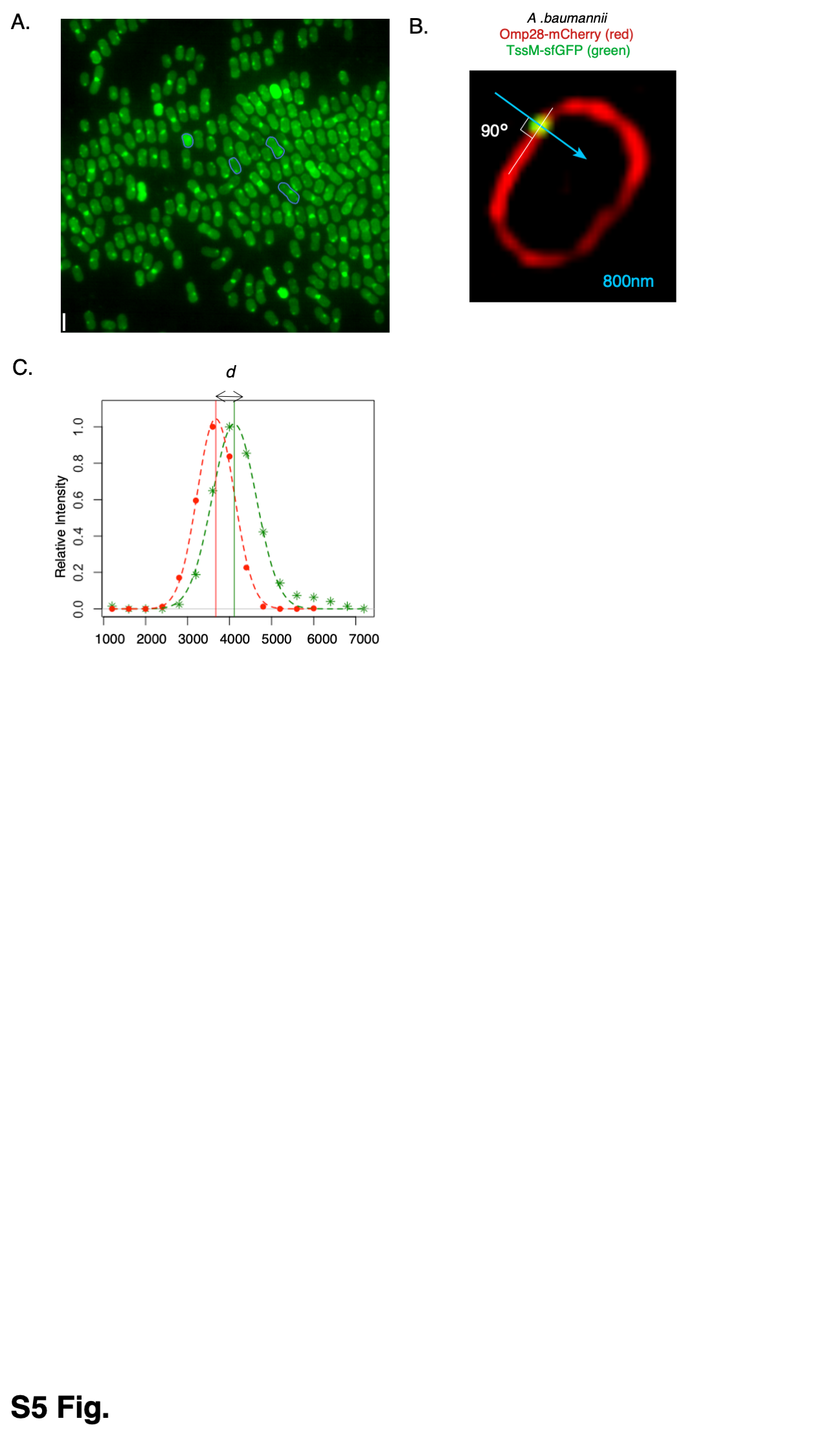

Supplement: S5 Fig — (A) Fluorescent clusters of the TssM protein observed in TIRF. Image associated with the Fig 5A. Projection of the average in a part of a full field of microscope acquisition. The corresponding cells have been circled in blue. Scale: 2 μm. (B-C) Schematic representation of the different stages leading to the identification of the subcellular position of the TssM foci. For each channel (sfGFP and mCherry) a line is drawn from which is created a Gaussian representing the intensity of fluorescence, depending on the position (right panel). The positions of the green foci (TssM) and the outer membrane (Omp28) are thus determined by the x axis of the maximum value of their respective Gaussian. The difference between the position of the green foci (x) and the red membrane (y) is calculated in order to determine the distance between the two marked regions (in absolute value) as well as the subcellular location of the C-terminal of TssM (negative results: the foci is “outside” the cell; positive results: the foci is “inside” the cell) (left panel). On the right panel, structured illumination microscopy (SIM) images of the C-terminal TssM-sfGFP regarding the Omp28-mCherry label. Scale = 0.1 μm. On the left panel, an example of an intensity profile (blue) is shown. Example of Gaussian representing the intensity sfGFP and mCherry fluorescence related to one green foci. (TIF) [file ppat.1011687.s006.tif]

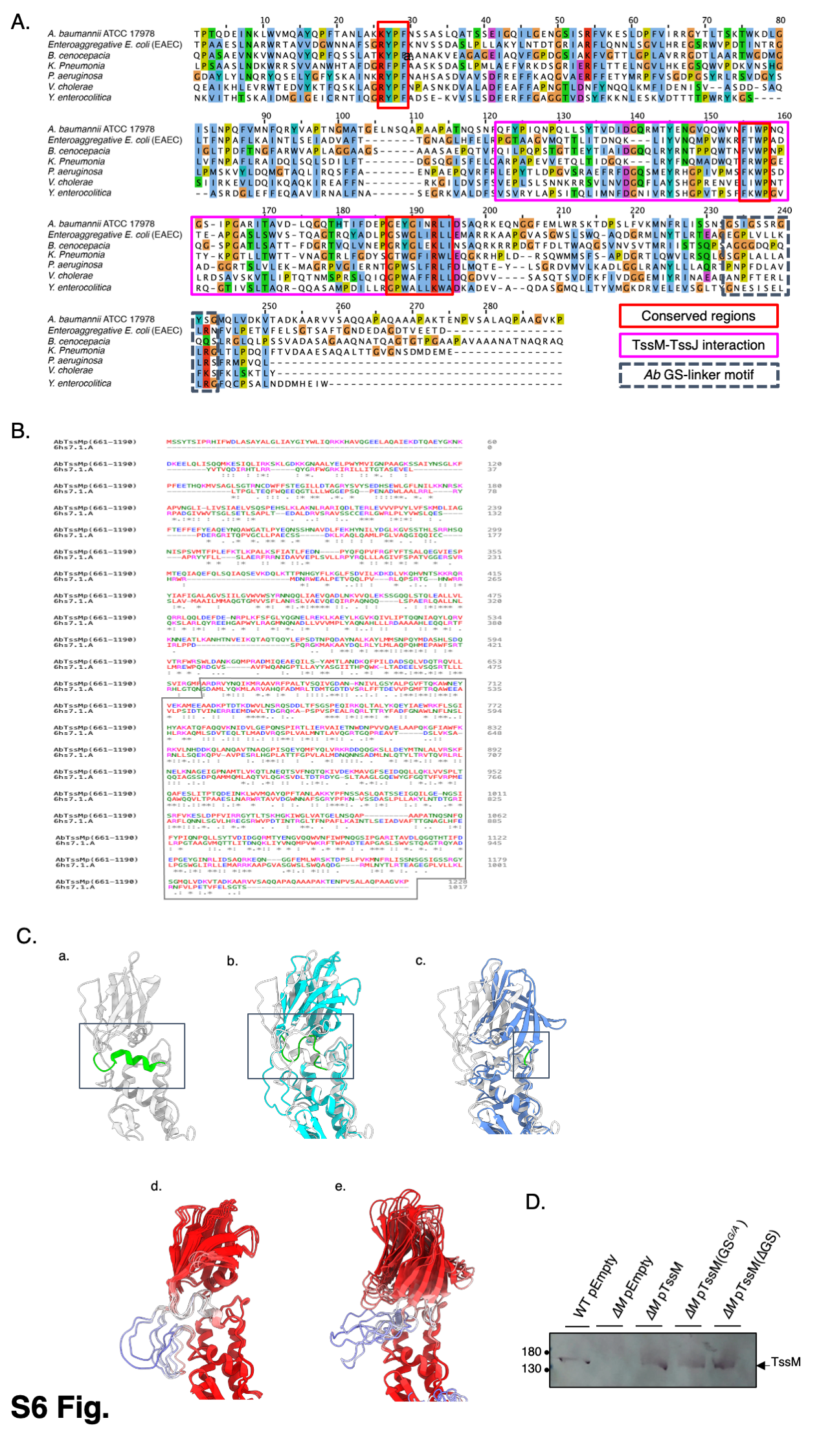

Supplement: S6 Fig — (A) MSA of A. baumannii and other proteobacterial species TssM C-terminal domain (from T987 to P1274 of A. baumannii TssM). Conserved regions were highlighted with a red square. The region of interaction between EAEC TssM and TssJ (PDB 4Y7O) was highlighted with a purple square. Residue conservation of the GxxGxxxGxxG motif found in the C-terminal domain of TssM for Acinetobacter was highlighted with grey dotted lines. (B) Multiple sequence alignment of A. baumannii TssM with the EAEC TssM (PDB 6hs7) to generate the structural model of A. baumannii TssM periplasmic domain. (C) Structural models for the TssM-CTD. (a) the structure of EAEC sequence. The region corresponding to the GS-linker of the Ab sequences is highlighted in green and boxed. (b,c) the structure of the A. baumannii sequence computed with AlphaFold (AF) 2.3.1 (cyan), and the A. baumannii-sequence with GS-linker deletion (blue) is aligned on the EAEC structure (gray), the GS-linker is colored in green and boxed. (d,e) 5 AF models for the Ab sequence colored using pLDDT score (red>90, blue<50). (D) Lower panel, western blot assays probing for TssM (WT and mutatants) production in whole cell lysates. The samples were subjected to denaturing 10%-polyacrylamide gel electrophoresis (PAGE) and immunodetected with synthetics polyclonal A. baumannii-TssM antibody. (TIF) [file ppat.1011687.s007.tif]
