# Supplementary material for: Acinetobacter type VI secretion system comprises a non-canonical membrane complex
Source: PLoS Pathog. 2023 Sep 28;19(9):e1011687. doi: 10.1371/journal.ppat.1011687 (PMC10564176; doi:10.1371/journal.ppat.1011687)
Supplement: S1 Table — (PDF) [file ppat.1011687.s008.pdf]

SUPPLEMENTAL TABLR S1. Strains, plasmids and oligonucleotides used in this study

Strains

Strains	Description and phenotype	Source
Acinetobacter baumannii		
ATCC 17978	Wild-type *Acinetobacter baumannii*	[1]
ATCC 17978:: *ΔtssM*	ATCC 17978 strain deleted of the *tssM (1302-1303)* gene	This paper
ATCC 17978:: sfGFP-*tssM*	*gfp-mut2* inserted downstream the start codon of *tssM* gene in ATCC 17978 strain	This paper
ATCC 17978:: *tssM-sfGFP*	*gfp-mut2* inserted upstream the stop codon of *tssM* gene in ATCC 17978 strain	This paper
ATCC 17978:: sfGFP-*tssL*	*gfp-mut2* inserted downstream the start codon of *tssL* gene in ATCC 17978 strain	This paper
ATCC 17978:: *tssM*-sfGFP :: mCherry-clpV	mCherry inserted downstream the start codon of *clpV (1307)* gene in ATCC 17978:: *tssM-sfGFP* strain	This paper
ATCC 17978:: *sfGFP-tssL*	*gfp-mut2* inserted upstream the stop codon of *tssL (1310)* gene in ATCC 17978 strain	This paper
ATCC 17978-*ΔtslA*	ATCC 17978 strain deleted of the *tslA (1292)* gene	This paper
ATCC 17978-*ΔtagX*	ATCC 17978 strain deleted of the *tagX (1311-12)* gene	This paper
ATCC 17978-*ΔtagN*	ATCC 17978 strain deleted of the *tagN (1305)* gene	This paper
ATCC 17978-*ΔtsmK*	ATCC 17978 strain deleted of the *tsmK (1301)* gene	This paper
ATCC 17978:: *omp28-mCherry:: gspC-sfGFP*	*m*Cherry inserted upstream the stop codon of *opm28 (2840)* gene and *gfp-mut2* inserted the stop codon of *gspC (0270)* in ATCC 17978 strain	This paper
ATCC 17978:: *tssM-sfGFP :: omp28-mCherry*	mCherry inserted downstream *opm28* gene in ATCC 17978:: *tssM- gfp-mut2*	This paper
ATCC 17978:: *tssM-m*Cherry	*m*Cherry inserted upstream the stop codon of *tssM* gene in ATCC 17978 strain	This paper
ATCC 17978:: *tssM*-Strep	Strep-Tag inserted upstream the stop codon of *tssM* gene in ATCC 17978 strain	This paper
E. coli K-12		

DH5α	F-, Δ(*argF*-l*ac*)U169, *phoA*, s*upE44*, Δ(lac*Z*)M15, *relA*, *endA*, *thi*, *hsdR*	New England Biolabs, Cat# C2987I
BL21(DE3)	*fhuA2 [lon] ompT gal (λ DE3) [dcm] ΔhsdS λ DE3 = λ sBamHIoΔEcoRI-B New England Biolabs int::(lacI::PlacUV5::T7 gene1) i21 Δnin5*	New England Biolabs, Cat# C2527I

Plasmids

Plasmids	Description	Source
Vector for protein over production		
pACYC-Duet	Expression vector, lacI, PT7, CM^{R}	Novagen Plasmid #71147-3
pCDF-Duet1	Expression vector, lacI, PT7, SM^{R}	Novagen Plasmid #71340-3
pRSF-Duet1	Expression vector, lacI, PT7, Kan^{R}	Novagen Plasmid #71341-3
pRSF-[^{STREP}TssL]	*tssL*-StrepII cloned into pRSF	This paper
pRSF-[^{HIS8}TssM^{FLAG}]	8xHIS-*tssM*-FLAG cloned into pRSF	This paper
pRSF-[^{STREP}TssL-^{HIS8}TssM^{FLAG}]	*tssL*-StrepII, 8xHIS-*tssM*-FLAG cloned into pRSF	This paper
pRSF-[^{STREP}TssL-^{HIS8}TssM_^{FLAG}-TsmK^{HA}]	*tssL*-StrepII, 8xHIS-*tssM*-FLAG, *tsmK*-HA cloned into pRSF	This paper
pCDF-[TsmK^{FLAG}]	*tsmK*-HA cloned into pRSF	This paper
pACYC-[^{FLAG}TagX^{VSVG}]	*tagX*-VSVG cloned into pACYC	This paper
pCDF-[TslA^{FLAG}]	*tslA*-FLAG cloned into pCDF	This paper
pACYC-[TagX^{STREP}]	*tagX*-STREPII cloned into pACYC	This paper
pACYC-[TsmK^{STREP}]	*tsmK*-STREPII cloned into pACYC	This paper
pRSF-[^{HIS6}TssM$_{930\text{-}1228}$^{FLAG}]	8xHIS-tssM$_{930\text{-}1228}$ -FLAG cloned into pRSF	This paper
pACYC-[TssM$_{930\text{-}1228}$^{STREP}]	tssM$_{930\text{-}1228}$ -STREPII cloned into pACYC	This paper
pACYC-[^{HIS6} TssM$_{930\text{-}1228}$ (ΔGS)^{FLAG}]	Mutagenesis of pRSF-[^{HIS6}TssM$_{930\text{-}1228}$^{FLAG}] to delete the Gly-Ser linker between the 1172 and 1179 residues	This paper
pRSF-[TssM$_{930\text{-}1228}$(ΔGS)^{STREP}]	Mutagenesis of pACYC-[TssM$_{930\text{-}1228}$^{STREP}] to delete the Gly-Ser linker between the 1172 and 1179 residues	This paper

pRSF-[$TssK^{His}_{Ab}$]	*Acinetobacter baumannii* tssK-StrepII cloned into pRSF-Duet1	This paper
pRSF-[$TssJ^{STREP}_{EAEC}$]	*EAEC 17-2* tssJ-StrepII cloned into pRSF-Duet1	This paper
pRSF-[TssM $ß_{EAEC}$]	*EAEC 17-2 tssM$_{972-1129}$* cloned into pRSF-Duet1	This paper
pRSF-[TssM $ß_{EAEC}$+$TssJ^{STREP}_{EAEC}$]	*EAEC 17-2 tssM$_{972-1129}$* and tssK-StrepII cloned into pRSF-Duet1	This paper
pRSF-[TssM $ß^{HIS}_{Ab}$]	*Acinetobacter* baumannii $tssM_{1054-1228}$ cloned into pRSF-Duet1	This paper
pRSF- [TssM $ß^{HIS}_{Ab}$(ΔGS)]	Mutagenesis of pRSF-[TssM $ß^{HIS}_{Ab}$] to delete the Gly-Ser linker between the 1172 and 1179 residues	This paper
Vector for chromosomal insertion		
pKD4	Kan^R cassette flanked by FRT recombination sites, used for chromosomal deletion	[2]
pKD4-mCherry (Cter)	*mCherry* gene cloned upstream the Kan^R cassette in pKD4, used for chromosomal insertion of *mCherry* (N-terminal mCherry)	[3]
pKD4-mCherry (Nter)	*mCherry* gene cloned downstream the Kan^R cassette in pKD4, used for chromosomal insertion of *mCherry* (C-terminal mCherry)	[4]
pKD4-sfGFP (Nter)	*gfp-mut2* (sf-gfp) gene cloned upstream the Kan^R cassette in pKD4, used for chromosomal insertion of gfp-mut2 (N-terminal GFP)	[5]
pKD4-sfGFP (Cter)	*gfp-mut2* (sf-gfp) gene cloned downstream the Kan^R cassette in pKD4, used for chromosomal insertion of gfp-mut2 (C-terminal GFP)	[4]
pKD4-strep (Cter)	*DNA sequence encoding STREPII tag* cloned upstream the Kan^R cassette in pKD4, used for chromosomal insertion of STREPII (N-terminal)	This paper
pATO4	pMMB67EH with RecAb system, Tetr	[6]
pATO3	pMMB67EH with FLP recombinase	[6]
For complementation experiement		
pVRL2	pVRL2 carrying the *araC*-P_{BAD} arabinose-inducible expression cassette; Gm^r	[7]
pVRL2-[$TssM(WT)^{His}$]	*tssM*-HIS cloned into pVRL2	This paper
pVRL2-[$TssM(GS)^{G/A\text{-}His}$]	Mutagenesis of the pVRL2-[$TssM(WT)^{His}$] to introduce a Gly→Ala substitution in position 1172, 1175 and 1179	This paper
pVRL2-[$TssM(ΔGS)^{His}$]	Mutagenesis of the pVRL2-[$TssM(WT)^{His}$] to delete the alpha helix between the 1172 and 1179 residues	This paper

pVRL2-[DsbA-TssMCter-peptide]^{HA}	Synthetic plasmid designed by Genecust that encompasses the DNA sequences encoding for an in frame *A. baumannii* DsbA signal sequence fused to TssM C-terminal TssM from amino acid 1166.	This paper - Genecust
pVRL1	E. coli-Acinetobacter species shuttle vector for general cloning purposes; Gm^{r}	[7]
pVRL1-[TsmK^{HA}]	*tsmK-HA* cloned into pVRL1	This paper

Oligonucleotides

**Oligonucleotides**	**Sequence (5'→3') & Description**
For biochemistry experiment	
pRSF-[^{STREP}TssL]	FW: ATACCATGGGCTGGAGCCACCCGCAGTTCGAAAAATCACAATCTACAGGTGCTCCTTCTTTATTTGACG<br>RV: AATGGATCCTCAAGGTAAGTAAATAGTAATATGTGCCTGCTCTTGTGGGGC<br><br>Insertion of the Ab ATCC 17978 TssL sequence into pRSF – Duet 1, N-terminal Strep-II epitope
pRSF-[^{HIS8}TssM^{FLAG}]	FW: ATAGGATCCAAGGAGATATACATATGCATCACCATCATCACCACCATCACTCTTCCTATACTTCAATTCCTCGCC<br>RV: AATGAGCTCTCACTTGTCATCGTCATCTTTATAATCTGGCTTAACTCCCGCAGCAGGTTGTGCTAGTGC<br><br>Insertion of the Ab ATCC 17978 TssM sequence into pRSF – Duet 1, C-terminal HISx8 and N-terminal FLAG epitope
pRSF-[^{STREP}TssL-^{HIS8}TssM^{FLAG}]	FW: ATAGGATCCAAGGAGATATACATATGCATCACCATCATCACCACCATCACTCTTCCTATACTTCAATTCCTCGCC<br>RV: AATGAGCTCTCACTTGTCATCGTCATCTTTATAATCTGGCTTAACTCCCGCAGCAGGTTGTGCTAGTGC<br><br>Insertion of the ^{HIS8}TssM^{FLAG} sequence into pRSF – ^{STREP}TssL
pRSF-[^{STREP}TssL-^{HIS8}TssM_^{FLAG}-TsmK^{HA}]	FW: ATACTGCAGAAGGAGATATACATATGATTAAAAAAATATTACTCTCATTCATCG<br>RV: AATGCGGCCGCTTAAGCGTAATCTGGAACATCGTATGGGTAGCTATTCAACACCATCGC<br><br>Insertion of Ab ATCC 17978 TsmK sequence into pRSF – ^{STREP}TssL-^{HIS8}TssM^{FLAG}, C-terminal HA epitope
pCDF-[TsmK^{FLAG}]	FW: ATACCATGGAAGGAGATATACATATGATTAAAAAAATATTACTCTCATTCATCG<br>RV: AATGGATCCTTACTTGTCATCGTCATCTTTATAATCGCTATTCAACACCATCGCATCATCTTCTG<br><br>Insertion of Ab ATCC 17978 TsmK sequence into pCDF-Duet, C-terminal FLAG epitope

pCDF-[TslA^{FLAG}]	FW: ATAAGATCTATGAAAGTTGTGTTGAAACG<br>RV: AATGGTACCTTACTTGTCATCGTCATCTTTATAATCTTTAATTAAAGGGTCAAAAGGCG<br><br>Insertion of the Ab ATCC 17978 TslA sequence into pCDF – Duet 1, C-terminal FLAG epitope
pACYC-[TagX^{STREP}]	FW: ATACATATGTGGAGCCACCCGCAGTTCGAAAAAATGATTTTTCTAATTTTCTTTTG<br>RV: AATCTCGAGTTAACTGATGTCATTTAAGCTTTCGGCAATCAGCTTTTCCGCC<br><br>Insertion of the Ab ATCC 17978 TagX sequence into pACYC – Duet 1, C-terminal STREP-II epitope
pACYC-[TagX^{VSVG}]	FW: ATACATATGATGATTTTTCTAATTTTCTTTTGTTTTGC<br>RV: AATCTCGAGTTATTTTCCTAATCTATTCATTTCAATATCTGTATAACTGATGTCATTTAAGCTTTCG<br><br>Insertion of the Ab ATCC 17978 TagX sequence into pACYC – Duet 1, C-terminal VSVG epitope
pACYC-[TsmK^{STREP}]	FW: ATACCATGGAAATTAAAAAAATATTACTCTCATTCATCGC<br>RV: AATGGATCCTTATTTTTCGAACTGCGGGTGGCTCCAGCTATTCAACACCATCGCATCATC<br><br>Insertion of Ab ATCC 17978 TsmK sequence into pCDF-Duet, C-terminal FLAG epitope
pRSF-[^{HIS6} TssM$_{930-1228}^{FLAG}$]	FW: ATTCCATGGccCACCATCATCACCACCATAAGATGGCGGTTGGTTTCAGTG<br>RV: AATGGATCCTCACTTGTCATCGTCATCTTTATAATCTGGCTTAACTCCCGCAGCAGG<br><br>Insertion of the Ab ATCC 17978 TssM$_{930-1228}$ into pRSF – Duet 1, C-terminal HISx6 and N-terminal FLAG epitope
pACYC-[TssM$_{930-1228}^{STREP}$]	FW: ATTCCATGGCCAAGATGGCGGTTGGTTTCAGTG<br>RV: AATGGATCCTCATTTTTCGAACTGCGGGTGGCTCCATGGCTTAACTCCCGCAGCAGG<br><br>Insertion of the Ab ATCC 17978 TssM$_{930-1228}$ into pRSF – Duet 1, N-terminal STREP-II epitope
pACYC-[TssM$_{930-1228}$ (ΔGS)^{STREP}]<br>or<br>pRSF-[^{HIS6} TssM$_{930-1228}$(ΔGS)^{FLAG}]	FW1: GTCTTATTAGTAGTAATTCAATGCAACTGGTTGACAAAGTAACTGCG<br>RV1: TTACTTTGTCAACCAGTTGCATTGAATTACTACTAATAAGACGGAAG<br><br>FW2: GCGACATCGTATAACGTTACTGGTTTCAC<br>RV2: GTGAAACCAGTAACGTTATACGATGTCGC<br><br>Slic mutagenesis from the pACYC-[TssM$_{930-1228}^{STREP}$] or the pRSF-[^{HIS6} TssM$_{930-1228}^{FLAG}$]
pRSF-[TssK$^{HIS}_{Ab}$]	FW : ATAGGATCCAGATTGGTAAAGAGATATGTTTAAAGCTGAAAAAGTCCTGTGG<br>RV : AATCTGCAGTTAAGCGTAATCTGGAACATCGTATGGGTAAGCGTTCATGACCGCAATCAGC<br><br>Restriction cloning, insertion of the Ab TssK sequence ATCC 17978 into pRSF – Duet, C-terminal His epitope
pRSF-[TssM ß$^{HIS}_{Ab}$]	FW1 : ATGAAAAAGACAGCTATCGCGATTGCAGTGGCACTGGCTGGTTTCGCTACCGTAGCGCAGGCCgcaaccaatcagtctaacttcc<br>RV1 : tcaGTGATGGTGGTGATGATGGTGATGtggcttaactcccgcagcagg<br>FW 2 : ATATTAGTTAAGTATAAGAAGGAGATATACATATGAAAAAGACAGCTATCGCG<br>RV 2 : ATCGCGTGGCCGGCCGATATCCAATTGAGATCTtcaGTGATGGTGGTGATGATGG

	Restriction cloning, insertion of the Ab TssMß sequence ATCC 17978 into pRSF – Duet, C-terminal His epitope
pRSF- [TssM $ß^{HIS}_{Ab}$(ΔGS)]	FW : TATTAGTAGTAATTCAGGATCTGGTATGCAACTGGTTGACAAAGTAACTGC<br>RV : GCAGTTACTTTGTCAACCAGTTGCATACCAGATCCTGAATTACTACTAATA<br><br>Mutagenesis, megapriming
pRSF-[$TssJ^{STREP}_{EAEC}$]	FW : GCAGTTCGAAAAATGACTCGAGAAGGAGATATACCATGGATTATAAAGATGACGATGACAAGAATAAACCTGTTATCTCCCGGGC<br>RV : ATGTATATCTCCTTTTATCCCTGCCCGGTAAGCCGTGCCACCTGGTCTG<br><br>Restriction free, megapriming
pRSF-[TssM $ß_{EAEC}$]	FW : ATACCATGGATGAAAAAGACAGCTATCGCGATTGCAGTGGCACTGGCTGGTTTCGCTACCGTAGCGCAGGCCGGTAACGCGGGGCTGCATTTTGAGC<br>RV : AATAAGCTTTCAGTCAGTCTCCTCCACGGTATCCCCGGCATCTTCGTCATTGCCTGTAAACGCTGAC<br><br>Restriction cloning
pRSF-[TssM $ß^{HIS}_{EAEC}$+$TssJ^{STREP}_{EAEC}$]	FW : ATACCATGGATGAAAAAGACAGCTATCGCGATTGCAGTGGCACTGGCTGGTTTCGCTACCGTAGCGCAGGCCGGTAACGCGGGGCTGCATTTTGAGC<br>RV : AATAAGCTTTCAGTCAGTCTCCTCCACGGTATCCCCGGCATCTTCGTCATTGCCTGTAAACGCTGAC<br><br>Restriction cloning
For strain construction	
A-del_*tssM*	FW: AATCCTAAAGCAATTATCGCCTTGTCATTGTTCGTGGCATTGATGGCGATTGTGTAGGCTGGAGCTGCTTC<br>RV: ATCAATGCTCCTCCTTGAATGTAGAGGTTAGTTTCCATGTCACATATGAATATCCTCCTTAGTTCCTATTCCG<br><br>A-primer, *tssM* deletion in *A. baumannii* ATCC 17978 strains
B-del_*tssM*	FW: AACTTAGAACGAGAATAGAATGCATACAATTTTAGGCTACTTGTGGCAGTACATCACGAATCCTAAAGCAATTATCGCC<br>RV: GAAGTCACCACGTGCAGGGCTTTTCCCGTAATACAGAGGAGTTGTTTTTAATGTATGCATCAATGCTCCTCCTTGAATG<br><br>B-primer, *tssM* deletion in *A. baumannii* ATCC 17978 strains
A-del_*tagN*	FW: TTTAAAACTATTTAGACAGACTTTTTTGGGTGAATAACAATGGCGATTGTGTAGGCTGGAGCTGCTTC<br>RV: ATCGTAATGTAAGGGCTAGCCATATTCTTCTCCTTATCCCTACATATGAATATCCTCCTTAGTTCCTATTCCG<br><br>B-primer, *tagN* deletion in *A. baumannii* ATCC 17978 strains
B-del_*tagN*	FW: CTGGTTTAGCAACGCTAGAACAATCTTTATGTCAACGCCAATTAAGTTTAAATCAAGGTTTAAAACTATTTAGACAGAC<br>RV: AATTAAAAATGCACTATTACCAGAAATGACTTGCCCTCCACCTGTCGTTGGGCATCCAATCGTAATGTAAGGGCTAGCC

	B-primer, *tagN* deletion in *A. baumannii* ATCC 17978 strains
A-del_*tsmK*	FW: AAAAGAAAGAGCAGGCTGCAAATCAAAGTCAAGATGATGATGCGATTGTGTAGGCTGGAGCTGCTTC<br>RV: ATATGTTCAGGAAAATGCATACTGAGCATAAAGCCATAAATCATATGAATATCCTCCTTAGTTCCTATTCCG<br><br>A-primer, *tsmK* deletion in *A. baumannii* ATCC 17978 strains
A-del_*tsmK*	FW: CATTACTTTGGTGATCTTGTCACCTTGGTTAATTTATAGGGTTTATAAAAGCTACAAAGAAAAGAAAGAGCAGGCTGC<br>RV: TGACACTCTGCACAAATGTATGTGCTGACACTGTATTTTCTCCAAAGACAAAAGGAACGATATGTTCAGGAAAATGC<br><br>B-primer, *tsmK* deletion in *A. baumannii* ATCC 17978 strains
A-del_*tslA*	FW: TTTCGGTAAAGCCTTGAATAAATAAGAAAACAGAGGAATGAGCGATTGTGTAGGCTGGAGCTGCTTC<br>RV: AAGCACTCACTTTATTACGCGTTGCGTACTGTTTTTTATAACATATGAATATCCTCCTTAGTTCCTATTCCG<br><br>A-primer, *tslA* deletion in *A. baumannii* ATCC 17978 strains
B-del_*tslA*	FW: TAAATTTTTTGAATATAAGGTTTTTATACTGATTTTTTAATATTCTAAAAATATTAACTTTTCGGTAAAGCCTTGAATA<br>RV: TTTAGGTTTGACAGCGGTGGCCTTTGCTGGACTTACTACTCGTTTATTTGATGTTCTAGAAGCACTCACTTTATTACGC<br><br>B-primer, *tslA* deletion in *A. baumannii* ATCC 17978 strains
A-del_*tagX*	FW: ATTACACGAAGTCCATTTGCTGTGAATTAGTTAAGTCATGGCGATTGTGTAGGCTGGAGCTGCTTC<br>RV: CCGTTTTACATGATCGCTTCCAATCAGGCTCCTAGGTTTACATATGAATATCCTCCTTAGTTCCTATTCCG<br><br>A-primer, *tagX* deletion in *A. baumannii* ATCC 17978 strains
B-del_*tagX*	FW: AAAGCTCCGGTTGAACAAGAGGCTGATGTGGAACCACCTAAAGCTTCGGTTGATATTAAGATTACACGAAGTCCATTTGC<br>RV: TTTCAATTTATACATGGGAAAAAGCTGGGTTGATTTCCCAGCTTGTCGATCACATGACTACCGTTTTACATGATCGCTTC<br><br>B-primer, *tagX* deletion in *A. baumannii* ATCC 17978 strains
A-del_*clpV*	FW: ATTATGATTTTAAGTATTAAATTATTTAAAAATATTGAGAGTAATGATGGCGATTGTGTAGGCTGGAGCTGCTTC<br>RV: ATACTCATCTAATTTTGGCTCTATTAAATTTTTAAACTTCTGATTTCAACATATGAATATCCTCCTTAGTTCCTATTCCG<br><br>A-primer, *clpV* deletion in *A. baumannii* ATCC 17978 strains
B-del_*clpV*	FW: TGATTGTAATGCTGGGTTATTTGTCGGATAAAGCTAAATTTTGGTAATATACGGGCATTATGATTTTAAGTATTAA<br>RV: TCACCACAAAGCAAGCTGTCATTAATGGGTTTAAGTAGTTCAGAAATATCAATACTCATCTAATTTTGGC<br><br>B-primer, *clpV* deletion in *A. baumannii* ATCC 17978 strains
A-del_*tssBC*	FW: GGCTAAGAGAGAAAGCGTACAAAAGAAGCTTCAGCGAATTCGACCTCCACGTGGCGATTGTGTAGGCTGGAGCTGCTTC<br>RV: TTTAGAACTTTCCTATTTTTAATTTAAAGTTATTCTTTAGCTGACCTTGATTACATATGAATATCCTCCTTAGTTCCTATTCCG<br><br>A-primer, *tssB and tssC* deletion in *A. baumannii* ATCC 17978 strains

B-del_*tssBC*	FW: TTCGCCTTTTGACCCTTTAATTAAATAATCCGGAATAGAATCTTATGGCTAAGAGAGAAAGCGTAC<br>RV: TCCATCAACTTTATATTTACCGCGAAACTCAACGTATATATCTTTCATTTAGAACTTTCCTATTTTT<br><br>B-primer, *tssB and tssC* deletion in *A. baumannii* ATCC 17978 strains
A-*sfGFP_tssM*	FW: TAAAGCAATTATCGCCTTGTCATTGTTCGTGGCATTGATGCGATTGTGTAGGCTGGAGCTGCTTCGAAGTTCCTATAC<br>RV: ATGCAGATGCTAAATCCCAAAATATATGGCGAGGAATTGAAGTATAGGAAGACCCTCCGCCGGCCGCTGC<br><br>A-primer, *sf-GFP* N-terminal insertion of *tssM* in *A. baumannii* ATCC 17978 strains
B-*sfGFP_tssM*	FW: TTAGAACGAGAATAGAATGCATACAATTTTAGGCTACTTGTGGCAGTACATCACGAATCCTAAAGCAATTATCGCCTTGTC<br>RV: TACGCTGAATTAGCCAATAAATTCCATAAGCGATGAGCCCTAAAGCATATGCAGATGCTAAATCCC<br><br>B-primer, *sf-GFP* N-terminal insertion of *tssM* in *A. baumannii* ATCC 17978 strains
A-*tssM_sfGFP*	FW: AAAACTGAAAATCCAGTTTCTGCACTAGCACAACCTGCTGCGGGAGTTAAGCCAGCAGCGGCCGGCGGAGGG<br>RV: TTTTTAATGTATGCATCAATGCTCCTCCTTGAATGTAGAGGTTAGTTTCCATGCATATGAATATCCTCCTTAGTTCCTATTCCGAAGTTCC<br><br>A-primer, *sf-GFP* C-terminal insertion of *tssM* in *A. baumannii* ATCC 17978 strains
B-*tssM_sfGFP*	FW: CGTTGTATCCGCACAGCAAGCACCAGCTCAGGCAGCTGCTCCTGCCAAAACTGAAAATCCAGTTTCTGC<br>RV: TAAGAAGTCACCACGTGCAGGGCTTTTCCCGTAATACAGAGGAGTTGTTTTTAATGTATGCATCAATGCT<br><br>B-primer, *sf-GFP* C-terminal insertion of *tssM* in *A. baumannii* ATCC 17978 strains
A-*sfGFP_tssL*	FW: TGATTGCGGTCATGAACGCTTAAAGATAAAGGAGAGAAAAATGCGATTGTGTAGGCTGGAGCTGCTTCGAAGTTCCTATAC<br>RV: TTTGCCCATCGTCAAATAAAGAAGGAGCACCTGTAGATTGTGACCCTCCGCCGGCCGCTGC<br><br>A-primer, *sf-GFP* N-terminal insertion of *tssM* in *A. baumannii* ATCC 17978 strains
B- *sfGFP_tssL*	FW: ATTCAGAAACGATTTGTATTTATGTTCCGGCTGGATTCCAAGATATCAGTATTGAGCTGATTGCGGTCATGAACGCT<br>RV: GATTAATTGCCTGTAACTTTACTTGGGACTGGCTATTATTATTCCCCCCTGTCCCAATTTGCCCATCGTCAAATAAA<br><br>B-primer, *sf-GFP* N-terminal insertion of *tssM* in *A. baumannii* ATCC 17978 strains
A- *clpV_mCherry*	FW: TGTGGCGAAGCCTGCTAAAAAACGGACTAGTAAAAAGTTGAAATCAGAAGTTGCAGCGGCCGGCGGAGGG<br>RV: ATTAATGGGTTTAAGTAGTTCAGAAATATCAATACTCATCATATGAATATCCTCCTTAGTTCCTATTCCGAAGTTCC<br><br>A-primer, *mCherry* C-terminal insertion of *clpV* in *A. baumannii* ATCC 17978 strains
B-*clpV_mCherry*	FW: GATCATGATTGATGCAAAAGATGACGAGATCCAATATTTACTAGATCCTGTGGCGAAGCCTGCTAAAAAACG<br>RV: CTTTCTTTATCTCGTGAAATTCATTAGAAAATGAATAATCCTCACCACAAAGCAAGCTGTCATTAATGGGTTTAAGTAGTTC<br><br>B-primer, *mCherry* C-terminal insertion of *clpV* in *A. baumannii* ATCC 17978 strains
A-*omp28_mCherry*	FW: GTACTGTAGTAGTTCAACCTGGTCAAGAAGCGGCAGCTCCTGCAGCAGCTCAAGCAGCGGCCGGCGGAGGG<br>RV: CCATAAAAAAAGCGACTCTAACGAGTCGCTTTTTTACTGTTCAAGAACTCAAACATATGAATATCCTCCTTAGTTCCTATTCCGAAGTTCC

	A-primer, *mCherry* C-terminal insertion of *omp28* in *A. baumannii* ATCC 17978 strains
B-*omp28_mCherry*	FW: AGGTCGTGCTATGAACCGTCGTGTATTCGCGACAATCACTGGTAGCCGTACTGTAGTAGTTCAACC<br>RV: GATTATGAATCAGGAGATTTACAAATGACCAAATATTTTAAAAATCGCCATAAAAAAAGCGACTCT<br><br>B-primer, *mCherry* C-terminal insertion of *omp28* in *A. baumannii* ATCC 17978 strains
For complementation experiement	
pVRL2-[TssM(WT)^{His}]	FW : ATACTCGAGATGTCTTCCTATACTTCAATTCC<br>RV : AATGCGGCCGCTCAGTGATGGTGGTGATGATGGTGATGTGGCTTAACTCCCGCAGCAGG<br><br>Insertion of the *A. baumannii* ATCC 17978 TssM sequence into pVRL2, C-terminal *8xHis* tag
1-pVRL2-[TssM$^{G/A-His}$]	FW1 : TCTTATTAGTAGTAATTCAGCAAGTATCGCTTCAAGCCGTGCTTACTCTGGTATGCAACTGGTTGAC<br>RV1 : ACCAGTTGCATACCAGAGTAAGCACGGCTTGAAGCGATACTTGCTGAATTACTACTAATAAGACGGAAGTTC<br><br>FW2 : ACATGGAAATGTCTGAAGCAACATGGTCGC<br>RV2 : GCGACCATGTTGCTTCAGACATTTCCATGT<br><br>Slic mutagenesis from the pVRL2-[TssM(WT)^{His}]
1-pVRL2-[TssM$^{\Delta GS}$]	FW1 : GTCTTATTAGTAGTAATTCAATGCAACTGGTTGACAAAGTAACTGCG<br>RV2 : TTACTTTGTCAACCAGTTGCATTGAATTACTACTAATAAGACGGAAG<br><br>FW : ACATGGAAATGTCTGAAGCAACATGGTCGC<br>RV : GCGACCATGTTGCTTCAGACATTTCCATGT<br><br>Slic mutagenesis from the pVRL2-[TssM(WT)^{His}]
pVRL1-[TsmK^{HA}]	FW1 : ATACTCGAGATGATTAAAAAAATATTACTC<br>RV1 : AATGCGGCCGCTTAAGCGTAATCTGGAACATCGTATGGGTAGCTATTCAACACCATCGC<br><br>Insertion of the Ab ATCC 17978 TsmK sequence into pVRL1, C-terminal HA-tag
pKD4-strep (Cter)	FW : GCAGCGGCCGGCGGAGGGTGGAGCCACCCGCAGTTCGAAAAATGAgtGTAGGCTGGAGCTGCTTCgaagttcc<br>RV : TCATTTTTCGAACTGCGGGTGGCTCCACCCTCCGCCGGCCGCTGCaatcgctcaagacgtgtaatgctgcaatc<br><br>Megapriming: insertion DNA sequence encoding STREPII tag in pKD4 (Kan)

Reference:

1. Weber BS, Miyata ST, Iwashkiw JA, Mortensen BL, Skaar EP, Pukatzki S, et al. Genomic and functional analysis of the type VI secretion system in Acinetobacter. PLoS One. 2013; 8(1):e55142. pmid: 23365692.
2. Datsenko KA, Wanner BL. One-step inactivation of chromosomal genes in Escherichia coli K-12 using PCR products. Proc Natl Acad Sci U S A. 2000; 97(12):6640-5. pmid: 10829079.
3. Zoued A, Durand E, Brunet YR, Spinelli S, Douzi B, Guzzo M, et al. Priming and polymerization of a bacterial contractile tail structure. Nature. 2016; 531(7592):59-63. pmid: 26909579.
4. Brunet YR, Zoued A, Boyer F, Douzi B, Cascales E. The Type VI Secretion TssEFGK-VgrG Phage-Like Baseplate Is Recruited to the TssJLM Membrane Complex via Multiple Contacts and Serves As Assembly Platform for Tail Tube/Sheath Polymerization. PLoS Genet. 2015; 11(10):e1005545. pmid: 26460929.
5. Durand E, Nguyen VS, Zoued A, Logger L, Péhau-Arnaudet G, Aschtgen MS, et al. Biogenesis and structure of a type VI secretion membrane core complex. Nature. 2015; 30;523(7562):555-60. pmid: 26200339.
6. Tucker AT, Nowicki EM, Boll JM, Knauf GA, Burdis NC, Trent MS, et al. Defining gene-phenotype relationships in Acinetobacter baumannii through one-step chromosomal gene inactivation. mBio. 2014; 5(4):e01313-14. pmid: 25096877.
7. Lucidi M, Runci F, Rampioni G, Frangipani E, Leoni L, Visca P. New Shuttle Vectors for Gene Cloning and Expression in Multidrug-Resistant Acinetobacter Species. Antimicrob Agents Chemother. 2018; 62(4):e02480-17. pmid: 29339383.
