# Supplementary material for: Acinetobacter type VI secretion system comprises a non-canonical membrane complex
Source: PLoS Pathog. 2023 Sep 28;19(9):e1011687. doi: 10.1371/journal.ppat.1011687 (PMC10564176; doi:10.1371/journal.ppat.1011687)
Supplement: S2 Table — (PDF) [file ppat.1011687.s009.pdf]

Bacteria	Acronym + Gene Cluster	Gene	GB accession no.	Reference
Acinetobacter baumannii	A. b	AsaB	AHB91901	[1]
		TssB	AHB91907	[1]
		TssK	AHB91894	[1]
		TssM	AHB91900	[1]
Aeromonas hydrophila	A. h	TssB	AJE36736	[2]
		TssK	AJE36729	[2]
		TssM	AJE36723	[2]
		TssJ	AJE36730	[2]
Agrobacterium Tumefaciens	A. t	TssB	AKC10282	[3]
		TssK	AKC10274	[3]
		TssM	AKC10272	[3]
Burkholderia cenocepacia	B. c 1	TssB	CDN61993	[4]
		TssK	CDN58852	[4]
		TssM	CDN62000	[4]
		TssJ	CDN62004	[4]
	B. c 2	TssB	CDN58855	[4]
		TssK	AIO54269	[4]

		TssM	CDN58865	[4]
		TssJ	CDN58853	[4]
Burkholderia Mallei	B. m 1	TssB	AIO54802	[5]
		TssK	AIO54269	[5]
		TssM	AIO54752	[5]
		TssJ	AIO54955	[5]
	B.m 2	TssB	AIO54309	[5]
		TssK	AIO54049	[5]
		TssM	AIO53403	[5]
		TssJ	AIO53789	[5]
	B.m 3	TssM	AIO52502	[5]
	B.m 4	TssB	AIO50864	[5]
		TssM	AIO53548	[5]
		TssJ	AIO54267	[5]
	B. m 5	TssB	AIO53430	[5]
		TssK	AIO54625	[5]
		TssM	AIO54885	[5]

		TssJ	AIO54963	[5]
Burkholderia pseudomallei	B. ps 1	TssB	CAH37543	[6]
		TssK	CAH37547	[6]
		TssM	CAH37549	[6]
		TssJ	CAH37546	[6]
	B. ps 2	TssB	CAH37618	[6]
		TssK	CAH37614	[6]
		TssM	CAH37612	[6]
		TssJ	CAH37615	[6]
	B. ps 3	TssB	CAH37973	[6]
		TssK	CAH37987	[6]
		TssM	CAH37989	[6]
		TssJ	CAH37986	[6]
	B. ps 4	TssB	CAH38969	[6]
		TssK	CAH38982	[6]
		TssM	CAH38984	[6]
		TssJ	CAH38981	[6]

	B. ps 5	TssB	CAH39580	[6]
		TssK	CAH39586	[6]
		TssM	CAH39584	[6]
		TssJ	CAH39587	[6]
	B. ps 6	TssB	CAH37118	[6]
		TssK	CAH37121	[6]
		TssM	CAH37108	[6]
Citrobacter freundii	C. f 1	TssB	AHY10367	[7]
		TssK	AHY10365	[7]
	C. f 2	TssB	AHY11618	[7]
		TssK	AHY11616	[7]
		TssM	AHY11614	[7]
Campylobacter Jejuni	C. j	TssB	AJK71142	[8]
		TssK	AJK71139	[8]
		TssM	AJK71136	[8]
		TssJ	AJK71140	[8]
Enteroaggregative Escherichia Coli	EAEC 1	TssB	CAU96112	[9]
		TssK	CAU96104	[9]

		TssM	CAU96099	[9]
		TssJ	CAU96105	[9]
	EAEC 2	TssB	CAU99327	[9]
		TssK	CAU99307	[9]
		TssM	CAU99303	[9]
		TssJ	CAU99309	[9]
	EAEC 3	TssB	CAU99394	[9]
		TssK	CAU99391	[9]
		TssM	CAU99370	[9]
		TssJ	CAU99362	[9]
Edwardsiella Tarda	E. t	TssB	ACY85264	[10]
		TssK	ACY85276	[10]
		TssM	ACY85278	[10]
		TssJ	ACY85275	[10]
Helicobacter hepaticus	H. h	TssB	AAP76845	[11]
		TssK	AAP76847	[11]
		TssM	AAP76849	[11]

		TssJ	AAP76846	[11]
Klebsiella Pneumonia	K. p 1	TssB	BAH63050	[12]
		TssK	BAH63077	[12]
		TssM	BAH63094	[12]
		TssJ	BAH63100	[12]
	K. p 2	TssK	BAH63996	[12]
		TssM	BAH63094	[12]
		TssJ	BAH63100	[12]
Pseudomonas Aeruginosa	P. a 1	TssB	AGY63037	[13]
		TssK	AGY66194	[13]
		TssM	AGY67240	[13]
		TssJ	AGY64572	[13]
	P. a 2	TssB	AGY68344	[13]
		TssK	AGY66073	[13]
		TssM	AGY67195	[13]
		TssJ	AGY67474	[13]
	P. a 3	TssB	AGY63622	[13]

		TssK	AGY63073	[13]
		TssM	AGY63404	[13]
Pseudomonas fluorescens	P. f	TssB	AIG01558	[14]
		TssK	AIG01564	[14]
		TssM	AIG01551	[14]
		TssJ	AIG01563	[14]
Paraburkholderia phymatum	P. p 1	TssB	ACC75041	[15]
		TssK	ACC75057	[15]
		TssM	ACC75059	[15]
	P. p 2	TssB	ACC75171	[15]
		TssK	ACC75167	[15]
		TssM	ACC75165	[15]
		TssJ	ACC75168	[15]
Rhizobium leguminosarum	R. l	TssB	AHF87021	[16]
		TssK	AHF87013	[16]
		TssM	AHF87011	[16]
Ralstonia solanacearum	R. s	TssB	CAD17894	[17]
		TssK	CAD17891	[17]

		TssM	CAD17914	[17]
		TssJ	CAD17892	[17]
Serratia Marcescens	S. m 1	TssB	AIA48002	[18]
		TssK	AIA48015	[18]
		TssM	AIA48017	[18]
		TssJ	AIA48014	[18]
	S. m 2	TssB	AIA48185	[18]
		TssK	AIA48190	[18]
		TssM	AIA48188	[18]
		TssJ	AIA48191	[18]
Salmonella Enterica Serovar Typhimurium	S. tm	TssB	AGQ68943	[19]
		TssK	AGQ68936	[19]
		TssM	AGQ68933	[19]
		TssJ	AGQ68937	[19]
Vibrio Cholerae	V. c	TssB	ACP07086	[20]
		TssK	ACP07093	[20]
		TssM	ACP07099	[20]
		TssJ	ACP07092	[20]

Vibrio parahaemolyticus	V. p 1	TssB	BAC62378	[21]
		TssK	BAC62384	[21]
		TssM	BAC62382	[21]
		TssJ	BAC62385	[21]
	V. p 2	TssB	BAC59665	[21]
		TssK	BAC59676	[21]
		TssM	BAC59671	[21]
		TssJ	BAC59675	[21]
Xanthomonas Citri	X. c	TssB	AAM38982	[22]
		TssK	AAM38956	[22]
		TssM	AAM38954	[22]
Xanthomonas Phaseoli	X. p	TssB	ATS30646	[23]
		TssK	ATS30666	[23]
Yersinia Enterocolitica	Y. e	TssB	AJJ27181	[24]
		TssK	AJJ27294	[24]
		TssM	AJJ29006	[24]
		TssJ	AJJ28527	[24]
Yersinia Pestis	Y. p 1	TssB	CAL19180	[25]

		TssK	CAL19194	[25]
		TssJ	CAL19193	[25]
	Y. p 2	TssB	CAL19645	[25]
		TssK	CAL19643	[25]
	Y. p 3	TssB	CAL20113	[25]
		TssK	CAL20115	[25]
		TssM	CAL20127	[25]
		TssJ	CAL20133	[25]
	Y. p 4	TssM	CAL21343	[25]
	Y. p 5	TssB	CAL21546	[25]
		TssK	CAL21542	[25]
		TssJ	CAL21543	[25]
	Y. p 6	TssB	CAL22294	[25]
		TssK	CAL22185	[25]
		TssM	CAL22191	[25]
		TssJ	CAL22184	[25]
Yersinia Pseudotuberculosis	Y. ps 1	TssB	ABS46925	[26]

		TssK	ABS46074	[26]
		TssM	ABS46522	[26]
		TssJ	ABS47321	[26]
	Y. ps 2	TssB	ABS46329	[26]
		TssK	ABS49416	[26]
	Y. ps 3	TssB	ABS46901	[26]
		TssK	ABS48268	[26]
		TssM	ABS48665	[26]
		TssJ	ABS49242	[26]
	Y. ps 4	TssB	ABS46608	[26]
		TssK	ABS48713	[26]
		TssM	ABS47333	[26]
		TssJ	ABS46683	[26]
	Y. ps 5	TssB	ABS49130	[26]
		TssK	ABS48245	[26]
		TssM	ABS47905	[26]
		TssJ	ABS46213	[26]

	Y. ps 6	TssB	ABS47113	[26]
		TssK	ABS49453	[26]
		TssJ	ABS47627	[26]

References

1. Wang X, Zhang Z, Hao Q, Gu W, Liu X, Guo B, Wu J, Yu J, Jing H, Xiao J, Yang,W. Complete Genome Sequence of Acinetobacter baumannii ZW85-1. Unpublished 2013

2. Pang M, Hu M, Kwok AHY, Jiang J, Leung FCC, Lu C, Liu Y. Genome sequencing of Aeromonas hydrophila J-1. Unpublished 2013

3. Huang YY, Cho ST, Lo WS, Wang YC, Lai EM, Kuo CH. Complete Genome Sequence of Agrobacterium tumefaciens Ach5. Genome Announc. 2015 Jun 4;3(3):e00570-15. PMID: 26044425

4. Carlier A, Bruggmann R, Agnoli K, Eberl L. Draft genome sequence of Burkholderia cenocepacia H111. Unpublished 2014

5. Daligault HE, Davenport KW, Minogue TD, Bishop-Lilly KA, Broomall SM, Bruce DC, Chain PS, Coyne SR, Frey KG, Gibbons HS, Jaissle J, Koroleva GI, Ladner JT, Lo CC, Munk C, Palacios GF, Redden CL, Rosenzweig CN, Scholz MB, Johnson SL. Whole-genome assemblies of 56 burkholderia species. Genome Announc. 2014 Nov 20;2(6):e01106-14. PMID: 25414490

6. Holden MT, Titball RW, Peacock SJ, Cerdeño-Tárraga AM, Atkins T, Crossman LC, et al. Genomic plasticity of the causative agent of melioidosis, Burkholderia pseudomallei. Proc Natl Acad Sci U S A. 2004; 101(39):14240-5. pmid: 15377794.

7. Conlan S, Korlach J, Thomas PJ, Mullikin J, Frank K,Palmore T. Segre JA. Whole genome sequencing of Citrobacter freundii. Unpublished 2014

8. Clark CG, Walker M, McCorrister SJ, Chong PM, Westmacott GR, Taboada EN. Proteomic comparison of four Campylobacter jejuni with reference to closed whole genome sequences. Unpublished 2014

9. Genoscope - Centre National de Sequencage : BP 191 91006 EVRY cedex - FRANCE. 2008

10. Wang Q, Yang M, Xiao J, Wu H, Wang X, Lv Y, et al. Genome sequence of the versatile fish pathogen Edwardsiella tarda provides insights into its adaptation to broad host ranges and intracellular niches. PLoS One. 2009; 4(10):e7646. PMID: 19865481

11. Suerbaum S, Josenhans C, Sterzenbach T, Drescher B, Brandt P, Bell M, et al. The complete genome sequence of the carcinogenic bacterium Helicobacter hepaticus. Proc Natl Acad Sci U S A. 2003;100(13):7901-6. PMID: 12810954.

12. Wu KM, Li LH, Yan JJ, Tsao N, Liao TL, Tsai HC, et al. Genome sequencing and comparative analysis of Klebsiella pneumoniae NTUH-K2044, a strain causing liver abscess and meningitis. J Bacteriol. 2009; 191(14):4492-501. PMID: 19447910.

13. Yin Y, Withers TR, Niles RM, Johnson SL, Yu HD. Draft Genome Sequences of Two Alginate-Overproducing Variants of Pseudomonas aeruginosa, PAO1-VE2 and PAO1-VE13. Genome Announce. 2013; 1(6):e01031-13. PMID: 24336371.

14. Dueholm MS, Danielsen HN, Nielsen PH. Complete Genome Sequence of Pseudomonas sp. UK4, a Model Organism for Studies of Functional Amyloids in Pseudomonas. Genome Announc. 2014; 2(5):e00898-14. PMID: 25212622.

15. Copeland A, Lucas S, Lapidus A, Glavina del Rio T, Bruce D, Goodwin L, et al. Complete sequence of plasmid1 of Burkholderia phymatum STM815. Unpublished 2008

16. Reeve W, Huntemann M, Han J, Chen A, Kyrpides N, et al. Mavromatis K, et al. DOE Joint Genome Institute - Direct Submission. Unpublished 2008

17. Salanoubat M, Genin S, Artiguenave F, Gouzy J, Mangenot S, Arlat M, et al. Genome sequence of the plant pathogen Ralstonia solanacearum. Nature. 2002; 415(6871):497-502. PMID: 11823852.

18. Li P, Kwok AH, Jiang J, Ran T, Xu D, Wang W, et al. Comparative genome analyses of Serratia marcescens FS14 reveals its high antagonistic potential. PloS one. 2015; 10(4), e0123061. PMID: 25856195.

19. Hoffmann M, Muruvanda T, Allard MW, Korlach J, Roberts RJ, et al. Complete Genome Sequence of a Multidrug-Resistant Salmonella enterica Serovar Typhimurium var. 5- Strain Isolated from Chicken Breast. Genome Announc. 2013; 1(6):e01068-13. Erratum in: Genome Announc. 2014 Apr 03;2(2):null. PMID: 24356834.

20. Feng L, Reeves PR, Lan R, Ren Y, Gao C, Zhou Z, et al. A recalibrated molecular clock and independent origins for the cholera pandemic clones. PloS one. 2008; 3(12), e4053. PMID: 19115014

21. Nasu H, Iida T, Sugahara T, Yamaichi Y, Park KS, Yokoyama K, et al. A filamentous phage associated with recent pandemic Vibrio parahaemolyticus O3:K6 strains. J Clin Microbiol. 2000; 38(6):2156-61. PMID: 10834969.

22. da Silva AC, Ferro JA, Reinach FC, Farah CS, Furlan LR, Quaggio RB, et al. Comparison of the genomes of two Xanthomonas pathogens with differing host specificities. Nature. 2002; 417(6887):459-63. PMID: 12024217.

23. Ruh M, Briand M, Bonneau S, Jacques MA, Chen NWG. Xanthomonas adaptation to common bean is associated with horizontal transfers of genes encoding TAL effectors. BMC Genomics. 2017; 18(1):670. PMID: 28854875.

24. Johnson SL, Daligault HE, Davenport KW, Jaissle J, Frey KG, Ladner JT, et al. Thirty-Two Complete Genome Assemblies of Nine Yersinia Species, Including Y. pestis, Y. pseudotuberculosis, and Y. enterocolitica. Genome Announc. 2015; 3(2):e00148-15. PMID: 25931590.
25. Parkhill J, Wren BW, Thomson NR, Titball RW, Holden MT, Prentice MB, et al. Genome sequence of Yersinia pestis, the causative agent of plague. Nature. 2001; 413(6855):523-7. PMID: 11586360.
26. Eppinger M, Rosovitz MJ, Fricke WF, Rasko DA, Kokorina G, Fayolle C, et al. The complete genome sequence of Yersinia pseudotuberculosis IP31758, the causative agent of Far East scarlet-like fever. PLoS Genet. 2007; 3(8):e142. PMID: 17784789.
